# Supplementary material for: SauCas9-based cell cycle-dependent genome editing via AAV delivery
Source: Mol Ther Adv. 2026 May 6;34(2):201751. doi: 10.1016/j.omta.2026.201751 (PMC13213762; doi:10.1016/j.omta.2026.201751)
Supplement: Document S1. Figures S1–S6, Tables S1–S3, and supplemental methods [file mmc1.pdf]

OMTA, Volume *34*

Supplemental information

SauCas9-based cell cycle-dependent genome editing via AAV delivery

Erina Matsugi, Kanae Kishi, Ayane Kishi, Kohei Nagase, Kiyomi Nigorikawa, and Wataru Nomura

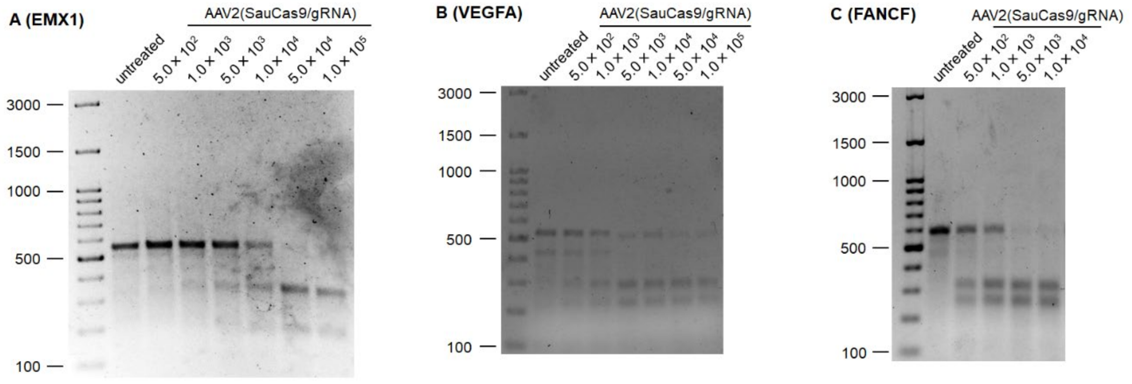

Figure S1. Functional titration of AAV2 for optimization of delivery. Indel efficiency occurred transduction of AAV2(SauCas9/gRNA) with variable viral titers was analyzed by T7E1 analysis on targets EMX1 (A), VEGFA (B), and FANCF (C).

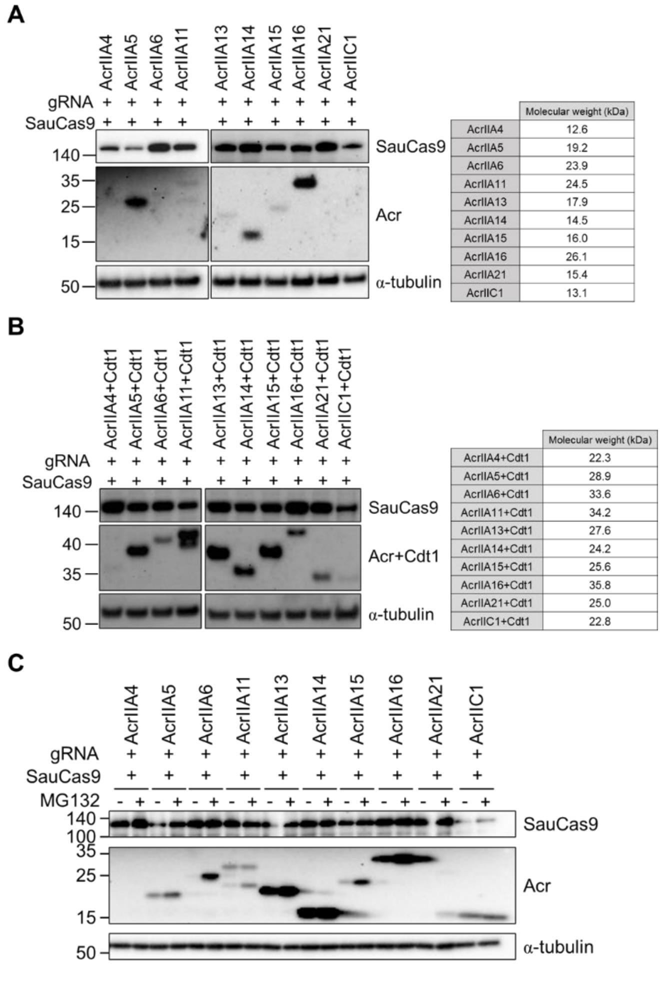

Figure S2. Protein expression analysis for Acr candidates (A), and their Cdt1 fusions (B). Cells were infected with the indicated AAVs at an MOI of 1.0×10^4 and collected for analysis at 72 h post-infection. (C) Effect of proteasome inhibition on Acr expression. The effect of the proteasome inhibitor MG132 (3 μM) on the expression of Acr candidates was evaluated.

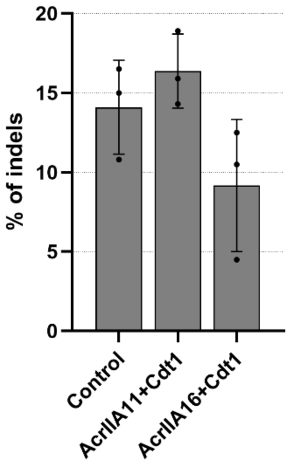

% of indels	Control	AcrIIA11+Cdt1	AcrIIA16+Cdt1
	16.5	15.9	12.5
	15	18.9	10.5
	10.8	14.3	4.5
Average	14.1	16.4	9.2
Standard Deviation	3.0	2.3	4.2
p-value compared with Control		0.6238	0.1861

Figure S3. Quantification of indel efficiency at the EMX1 locus for samples in Figure 4D. The "Control" group represents cells co-transduced with AAV2(SauCas9) and AAV2(gRNA/HDR template). The "AcrIIA11+Cdt1" and "AcrIIA16+Cdt1" groups represent cells co-transduced with AAV2(SauCas9) and either AAV2(AcrIA11+Cdt1/gRNA/HDR template) or AAV2(AcrIIA16+Cdt1/gRNA/HDR template), respectively. Total editing efficiency was quantified by Sanger sequencing followed by TIDER analysis. The indel mutation rate was determined by subtracting the HDR efficiency from the total editing efficiency calculated via the TIDER program. Statistical significance was analyzed by one-way ANOVA followed by Dunnett's post-hoc test.

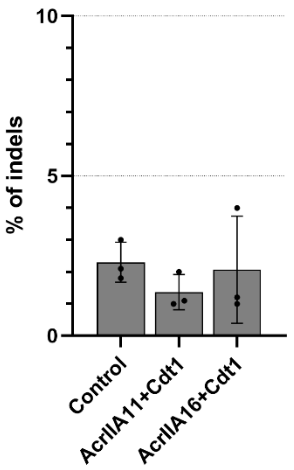

% of indels	Control	AcrIIA11+Cdt1	AcrIIA16+Cdt1
	2.1	2	1.2
	1.8	1	4
	3	1.1	1
Average	2.3	1.4	2.1
Standard Deviation	0.6	0.6	1.7
p-value compared with Control		0.5059	0.9507

Figure S4. Quantification of indel efficiency at the EMX-1 off-target site using the genomic DNA from experiments in Figure 4D. Indel efficiency was quantified by Sanger sequencing followed by TIDE analysis. Off-target locus was amplified by PCR using primers: forward (5'-AACTCCGTCTGTTGATCGGT-3') and reverse (5'-TGCTAGGCTTGAATAACCTGGA-3'). The "Control" group represents cells transduced with AAV2(SauCas9) and AAV2(gRNA/HDR template). The "AcrIIA11+Cdt1" and "AcrIIA16+Cdt1" groups represent cells transduced with AAV2(SauCas9) and either AAV2(AcrIA11+Cdt1/gRNA/HDR template) or AAV2(AcrIIA16+Cdt1/gRNA/HDR template), respectively. Statistical significance was analyzed by one-way ANOVA followed by Dunnett's post-hoc test.

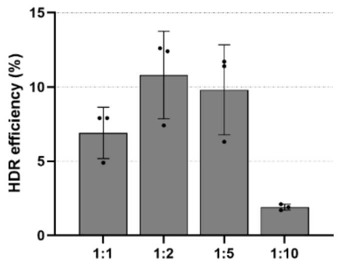

HDR	1:1	1:2	1:5	1:10
	7.9	12.6	11.7	2.1
	7.9	12.4	6.3	1.7
	4.9	7.4	11.4	1.9
Average	6.9	10.8	9.8	1.9
Standard Deviation	1.7	2.9	3.0	0.2
p-value compared with (1:1)		0.16	0.3388	0.0675

Figure S5. Dose-dependent effects of AAV2 carried HDR template on HDR efficiency. The MOI of AAV2(SauCas9) was held constant at $1.0×10^4$, whereas the MOI of AAV2(gRNA/HDR template) was increased as indicated: 1:1 ($1.0×10^4$), 1:2 ($2.0×10^4$), 1:5 ($5.0×10^4$), and 1:10 ($1.0×10^5$). Statistical significance was analyzed by one-way ANOVA followed by Dunnett's post-hoc test.

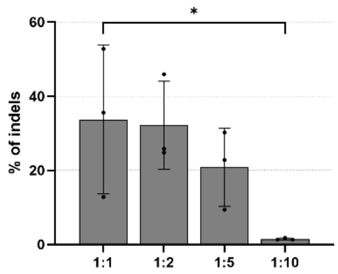

% of indels	1:1	1:2	1:5	1:10
	35.6	25.8	9.4	1.3
	52.8	45.9	22.8	1.8
	12.8	24.8	30.2	1.3
Average	33.7	32.2	20.8	1.5
Standard Deviation	20.1	11.9	10.5	0.3
p-value compared with (1:1)		0.9974	0.5	0.0368

Figure S6. Quantification of indel efficiency at the EMX1 locus for samples in Figure 4E. The MOI of AAV2(SauCas9) was held constant at $1.0×10^4$, whereas the MOI of AAV2(AcrIIA11+Cdt1/gRNA/HDR template) was increased as indicated: 1:1 ($1.0×10^4$), 1:2 ($2.0×10^4$), 1:5 ($5.0×10^4$), and 1:10 ($1.0×10^5$). Total editing efficiency was quantified by Sanger sequencing followed by TIDER analysis. The indel mutation rate was determined by subtracting the HDR efficiency from the total editing efficiency calculated via the TIDER program. Statistical significance was analyzed by one-way ANOVA followed by Dunnett's post-hoc test (*$p < 0.05$).

Table S1 Sequences of synthetic oligonucleotides coding Acr genes.

Anti-CRISPR	Synthetic gene sequence
	SV40NLS, anti-CRISPR, HA
AcrIIA1	CCGAAGAAGAAACGGAAGGTAGGTGGCGGCTTTAATAAGGCTGAAATTATGAAACAAGCTTGGAATTGGTTCAACGATAGCAACATCACGATAAAGCTGTTGGACGAGTTTCTGAAGAAACACGATCTCACACGGTATCAGCTCTCCAAGTTGACAGGGATTAGCCAGAATACTCTCAAAGACCAGAATGAGAAACCTCTCAACAAGTACACCGTCAGTATCCTGAGATCACTGAGTCTGATTAGCGGACTTTCTGTGTCTGATGTGCTGTTCGAACTGGAAGATATCGAGAAGAATTCCGACGATCTTGCTGGCTTCAAACATCTGCTCGACAAATACAAACTGAGCTTTCCAGCACAAGAGTTTGAGCTGTATTGCCTGATAAAGGAGTTCGAATCAGCGAATATCGAGGTACTGCCCTTTACCTTCAACAGGTTCGAGAACGAAGAACACGTTAACATCAAGAAGGACGTCTGTAAAGCCCTGGAAAATGCCATTACTGTGCTGAAAGAGAAGAAGAACGAACTTTTGGGTGGCTCTTACCCCTACGACGTGCCCGACTACGCC
AcrIIA2	CCGAAGAAGAAACGGAAGGTAGGTGGCGGCTTTAATAAGGCTGAAATTATGACGCTTACGCGGGCGCAGAAGAAATACGCGGAGGCAATGCACGAGTTTATCAACATGGTGGATGACTTCGAGGAATCCACACCGGACTTTGCGAAGGAGGTTTTGCACGATAGTGATTACGTAGTCATCACTAAGAACGAAAAGTACGCGGTTGCACTGTGCTCCTTGTCAACAGATGAGTGTGAGTACGATACGAATTTGTATCTTGATGAAAAGTTGGTAGACTATAGCACTGTGGACGTTAACGGAGTCACCTACTACATAAACATCGTTGAGACCAACGATATTGATGATCTTGAGATAGCCACAGATGAAGACGAGATGAAATCAGGAAACCAGGAAATAATCCTTAAGTCTGAACTTGGTGGCTCTTACCCCTACGACGTGCCCGACTACGCC
AcrIIA3	CCGAAGAAGAAACGGAAGGTAGGTGGCGGCTTTAATAAGGCTGAAATTATGTTCAACAAAGCGGAAATAATGAAACAAGCCTGGAATTGGTTTAACGATTCCAATATATGGCTCTCCGATATTGAGTGGGTGTCATACACAGATAAAGAGAAGTCTTTCAGCGTCTGTCTGAAGGCAGCTTGGAGTAAAGCCAAAGAGGAAGTTGAAGAATCTAAAAAGGAGTCTAAACATATTGCAAAATCAGAGGAGCTTAAAGCCTGGAACTGGGCCGAAAGAAAACTTGGCCTCCACTTCAATATTAGCGATGACGAAAAATTTACTTCCGTTAAAGATGAAACGAAGATAAACTTTGGCCTGTCTGTGTGGGCATGCGCCATGAAAGCCGTGAAACTCCATAATGATCTCTTTCCTCAGACCGCCGCAGGTGGCTCTTACCCCTACGACGTGCCCGACTACGCC
AcrIIA4	CCGAAGAAGAAACGGAAGGTAGGTGGCGGCAATATAAATGACCTCATAAGAGAGATAAAGAACAAAGATTACACAGTCAAGCTGAGCGGGACTGACTCCAACAGTATCACTCAACTGATTATCCGCGTCAATAATGATGGGAACGAGTACGTTATTAGTGAAAGCGAAAATGAGTCAATAGTCGAGAAGTTTATCTCCGCTTTTAAGAACGGTTGGAATCAGGAATATGAGGACGAGGAAGAATTTTATAATGACATGCAGACAATAACGCTGAAAAGTGAGCTGAATGGTGGCTCTTACCCCTACGACGTGCCCGACTACGCC
AcrIIA5	CCGAAGAAGAAACGGAAGGTAGGTGGCGGCGCATACGGAAAAAGCAGATACAACTCATACAGGAAACGGAACTTCAGCATTAGCGACAATCAGCGGAGAGAATACGCAAAAAAGATGAAGGAGCTGGAGCAGGCCTTCGAGAACCTGGACGGCTGGTACCTGAGCTCCATGAAGGACTCCGCCTACAAGGATTTTGGCAAGTATGAGATCAGGCTGTCTAACCACAGCGCCGACAATCGGTATCACGATCTGGAGAACGGCAGACTGATCGTGAATGTGAAGGCCTCTAAGCTGAACTTCGTGGACATCATCGAGAATAAGCTGGGCAAGATCATCGAGAAGATCGATACCCTGGACCTGGATAAGTACCGGTTTATCAATGCTACAAAACTGGAGAGAGATATTAAGTGCTATTACAAAGGATACAAAACCAAAAAGGATGTGATTGGTGGCTCTTACCCCTACGACGTGCCCGACTACGCC
AcrIIA6	CCGAAGAAGAAACGGAAGGTAGGTGGCGGCAAGATTAACGATGACATTAAGGAACTGATTCTGGAGTATATGAGCCGCTATTTCAAATTCGAGAACGACTTTTACAAACTGCCTGGCATCAAGTTCACCGACGCCAACTGGCAGAAGTTTAAGAATGGCGGCACAGATATCGAGAAGATGGGAGCAGCAAGGGTGAACGCAATGCTGGACTGCCTGTTCGACGATTTTGAGCTGGCCATGATCGGCAAGGCCCAGACCAACTACTATAACGACAATTCCCTGAAGATGAATATGCCCTTCTACACATACTACGATATGTTCAAGAAGCAGCAGCTGCTGAAGTGGCTGAAGAACAATCGGGACGATGTGATCGGCGGCACCGGCAGAATGTATACAGCCTCTGGCAACTACATCGCCAATGCCTATCTGGAGGTGGCCCTGGAGAGCTCCTCTCTGGGCAGCGGCTCCTACATGCTGCAGATGCGGTTCAAGGATTATAGCAAGGGCCAGGAGCCTATCCCAAGTGGAAGACAGAACAGACTGGAGTGGATTGAAAACAACCTGGAGAACATCAGAGGTGGCTCTTACCCCTACGACGTGCCCGACTACGCC
AcrIIA11	CCGAAGAAGAAACGGAAGGTAGGTGGCGGCGCAGATATGACGCTTCGCCAGTTCTGCGAGCGATATCGCAAGGGTGACTTCCTCGCAAAGGATCGAGAAACTCAAATCGAGGCAGGTTGGTACGATTGGTTTTGTGATGACAAAGCCTTGGCGGGCCGATTGGCAAAAATCTGGGGGATTTTGAAGGGGATAACCTCAGATTATATCTTGGATAACTACCGCGTATGGTTCAAAAACAACTGTCCAATGGTAGGACCACTGTACGACGATGTACGCTTCGAACCGCTTGATGAAGAACAGCGAGATGAGCTCTACTTCGGCGTCGCAATCGACGATAAGAGGAGGGAAAAGAAATACGTCATATTCACTGCTCGAAATGACTATGAAAACGAGTGTGGTTTCAACAACGTGAGAGAAGTACGCCAATTTATAAATGGATGGGAAGACGAATTGAAGAACGAAGAGTTCTATAAGGCTAGGGAGAAAAAACGGCAAGAAATGGAAGAAGCCAATAACAAATTCGCAGAAATAATGCAACGGGCCGATGAGATATTGTGGAACCTGAAAGAGGACGGTGGCTCTTACCCCTACGACGTGCCCGACTACGCC
AcrIIA13	CCGAAGAAGAAACGGAAGGTAGGTGGCGGCATGAAGTCCGTGAAGTACATCAGCAACATGAGCAAGCAAGAGAAAGGCTACCGGGTGTACGTGAACGTGGTCAACGAGGATACCGACAAGGGCTTTCTGTTCCCCAGCGTGCCCAAAGAAGTGATCGAGAACGACAAGATCGACGAGCTGTTCAACTTCGAGCACCACAAGCCTTACGTGCAGAAGGCCAAGAGCAGATACGACAAGAACGGCATCGGCTACAAGATCGTGCAGCTGGACGAGGGCTTCCAGAAGTTCATCGAGCTGAACAAAGAAAAGATGAAGGAAAACCTGGACTACGGTGGCTCTTACCCCTACGACGTGCCCGACTACGCC
AcrIIA14	CCGAAGAAGAAACGGAAGGTAGGTGGCGGCATGAAGTCCGTGAAGTACATCAGCAACATGAGCAAGCAAGAGAAAGGCTACCGGGTGTACGTGAACGTGGTCAACGAGGATACCGACAAGGGCTTTCTGTTCCCCAGCGTGCCCAAAGAAGTGATCGAGAACGACAAGATCGACGAGCTGTTCAACTTCGAGCACCACAAGCCTTACGTGCAGAAGGCCAAGAGCAGATACGACAAGAACGGCATCGGCTACAAGATCGTGCAGCTGGACGAGGGCTTCCAGAAGTTCATCGAGCTGAACAAAGAAAAGATGAAGGAAAACCTGGACTACGGTGGCTCTTACCCCTACGACGTGCCCGACTACGCC
AcrIIA15	CCGAAGAAGAAACGGAAGGTAGGTGGCGGCGTGGATACCTGGATCGTGTACAGAGGCAGAACCGCCGACATGAACAAGAGCTATATCGCCGAGGGCAGCACCTACGAGGAAGTGTACAACAACTTCGTGGATAAGTACGGCTACGACGTGCTGGACGAGGACATCTACGAGATCCAGCTGCTGAAGAAGAACGGCGAGAACCTGGACGACTACGATGTGGACAGCGACGGCATCAACAACTACGACAAGCTGGATGAGTTCCGCGAGAGCGACTACGTGGACCTGGAAGATTACGACTACAGAGAGCTGTTCGAGAACAGCAGCAGCCAGGTGTACTACCACGAGTTCGAGATCACCCACGAGGGTGGCTCTTACCCCTACGACGTGCCCGACTACGCC
AcrIIA16	CCGAAGAAGAAACGGAAGGTAGGTGGCGGCATGGGCTACATCGGCACCAAGAGAAGCGAGAGAAGCCAGGACGCCATCGAGGATTACGAGGTGCCCCTGAACCACTTCAACAAGGACCTGATCCAGGCCTTCATCGACGAGAACGAGGCCTACGACACCCTCAAGACCAAGAAAGTGCGGCTGTGGAAGTTCGTGGCCCCTAGAGCTGGTGCCACAAGCTGGCATCACACCGGCACCTACTACAACAAGACCGACCACTACAGCCTGGAAAAGGTGGCCGATGAGCTGCTGCAGAATGGCGACGAGTGGGAAGAACAGTTCAAGGCCTACGTGAAAGAGGAACAAGAGACAGCTACCAGCGAGCCCGTGTTCCTGAGCGTGATCAAGGTGCAGATCTGGGGCGGCAGCATGAAGAGGCCTAAACTCGTGGGACACGAGGTGGTCATGGGCGTGAAGAAAGAAGGATGGCTGCACGCCGTGTCCAAGGCCACACAGAGCAAGTACAAGCTGAGCGCCAACAAGGTGGAAATGCAGAAGCACTACTCCCTCGAGGACTACAGCGCCCTGACCAAGGACTTCCCCGAGTTCAAAGCCCAGAAGCGGGCCATCAACAAGAAAATGAAGGAAATGTACAACGGTGGCTCTTACCCCTACGACGTGCCCGACTACGCC
AcrIIA21	CCGAAGAAGAAACGGAAGGTAGGTGGCGGCATGGACTACGACAACGAGAACTATCTGATCCCCAAGATCCTGCTGCAGGACGACTTCTACAGCAGCCTGAGCGCCAAGGACATCCTGGTGTATGCCGTGCTGAAGGACAGACAGATCGAGGCCCTGGAAAAAGGCTGGATCGACACCGACGGCAGCATCTACCTGAACTTCAAGCTGATCGAGCTGGCCAAGATGTTCAGCTGCAGCCGGACCACCATGATCGACGTGATGCAGCGGCTGGAAGAAGTGAACCTGATCGAGCGCGAGCGCGTGGACGTGTTCTACGGCTACAGCCTGCCTTACAAGACCTACATCAACGAGGTGGGTGGCTCTTACCCCTACGACGTGCCCGACTACGCC
AcrIIC1	CCGAAGAAGAAACGGAAGGTAGGTGGCGGCATGAAGGAAGTGTTCAAGCTGAAGCCCGAGCTGGTCACCTACAAAGGCTGTGGATGGGCCCTCGCCTGCATCAAGGATGGCGAGATCATCGACCTGACCTACGTCAGAGATCTGGGCATCGAGGAATACGACGAGAACTTCGACGGCCTGGAACCTGAGATCATCTACTACGACGTGGTGGCCAGCCAGGCCTGTAAAGAGGTGGCCTACAGATACGAGGAAATGGGCGAGTTCACCTTCGGCCTGTGCAGCTGCTGGGAGTTCAACGTGATGGGTGGCTCTTACCCCTACGACGTGCCCGACTACGCC

Tables S2 Amino acid sequences of Acr/Acr-Cdt1 constructs, their molecular weight, and accession numbers.

Acr	Molecular weight	Amino acid sequence
AcrIIA4	12609.9	MGGPKKKRKVGGGNINDLIREIKNKDYTVKLSGTDSNSITQLIIRVNNDGNEYVISESENESIVEKFISAFKNGWNQEYEDEEEFYNDMQTITLKSELNGGSYPYDVPDYA
AcrIIA5	19184.8	MGGPKKKRKVGGGAYGKSRYNSYRKRNFSISDNQRREYAKKMKELEQAFENLDGWYLSSMKDSAYKDFGKYEIRLSNHSADNRYHDLENGRLIVNVKASKLNFVDIIENKLGKIIEKIDTLDLDKYRFINATKLERDIKCYYKGYKTKKDVIGGSYPYDVPDYA
AcrIIA6	23876.1	MGGPKKKRKVGGGKINDDIKELILEYMSRYFKFENDFYKLPGIKFTDANWQKFKNGGTDIEKMGAARVNAMLDCLFDDFELAMIGKAQTNYYNDNSLKMNMPFYTYYDMFKKQQLLKWLKNNRDDVIGGTGRMYTASGNYIANAYLEVALESSSLGSGSYMLQMRFKDYSKGQEPIPSGRQNRLEWIENNLENIRGGSYPYDVPDYA
AcrIIA11	24531.5	MGGPKKKRKVGGGADMTLRQFCERYRKGDFLAKDRETQIEAGWYDWFCDDKALAGRLAKIWGILKGITSDYILDNYRVWFKNNCPMVGPLYDDVRFEPLDEEQRDELYFGVAIDDKRREKKYVIFTARNDYENECGFNNVREVRQFINGWEDELKNEEFYKAREKKRQEMEEANNKFAEIMQRADEILWNLKEDGGSYPYDVPDYA
AcrIIA13	17920.8	MGGPKKKRKVGGGMEVMNKSIEIKDQNNIVLIDSLGQFFTDIENDNNGRYNIDYVLLNEVEHDNGNTYYEVGMYRTEEVPFSDKVTQDNVELLEDKWLQIDQQGESYVESIFFENEEDAREYIKLVLKGHETFEETAKAIGVIKGGSYPYDVPDYA
AcrIIA14	14484.5	MGGPKKKRKVGGGMKSVKYISNMSKQEKGYRVYVNVVNEDTDKGFLFPSVPKEVIENDKIDELFNFEHHKPYVQKAKSRYDKNGIGYKIVQLDEGFQKFIELNKEKMKENLDYGGSYPYDVPDYA
AcrIIA15	15954.1	MGGPKKKRKVGGGVDTWIVYRGRTADMNKSYIAEGSTYEEVYNNFVDKYGYDVLDEDIYEIQLLKKNGENLDDYDVDSDGINNYDKLDEFRESDYVDLEDYDYRELFENSSSQVYYHEFEITHEGGSYPYDVPDYA
AcrIIA16	26087.6	MGGPKKKRKVGGGMGYIGTKRSERSQDAIEDYEVPLNHFNKDLIQAFIDENEAYDTLKTKKVRLWKFVAPRAGATSWHHTGTYYNKTDHYSLEKVADELLQNGDEWEEQFKAYVKEEQETATSEPVFLSVIKVQIWGGSMKRPKLVGHEVVMGVKKEGWLHAVSKATQSKYKLSANKVEMQKHYSLEDYSALTKDFPEFKAQKRAINKKMKEMYNGGSYPYDVPDYA
AcrIIA21	15362.6	MGGPKKKRKVGGGMDYDNENYLIPKILLQDDFYSSLSAKDILVYAVLKDRQIEALEKGWIDTDGSIYLNFKLIELAKMFSCSRTTMIDVMQRLEEVNLIERERVDVFYGYSLPYKTYINEVGGSYPYDVPDYA
AcrIIC1	13143.0	MGGPKKKRKVGGGMKEVFKLKPELVTYKGCGWALACIKDGEIIDLTYVRDLGIEEYDENFDGLEPEIIYYDVVASQACKEVAYRYEEMGEFTFGLCSCWEFNVMGGSYPYDVPDYA
AcrIIA4+Cdt1	22293.8	MGGPKKKRKVGGGNINDLIREIKNKDYTVKLSGTDSNSITQLIIRVNNDGNEYVISESENESIVEKFISAFKNGWNQEYEDEEEFYNDMQTITLKSELNGGSYPYDVPDYALEPSPARPALRAPASATSGSRKRARPPAAPGRDQARPPARRRLRLSVDEVSSPSTPEAPDIPACPSPGQKIKKSTPAAGQPPHLTSAQDQDTI
AcrIIA5+Cdt1	28868.7	MGGPKKKRKVGGGAYGKSRYNSYRKRNFSISDNQRREYAKKMKELEQAFENLDGWYLSSMKDSAYKDFGKYEIRLSNHSADNRYHDLENGRLIVNVKASKLNFVDIIENKLGKIIEKIDTLDLDKYRFINATKLERDIKCYYKGYKTKKDVIGGSYPYDVPDYALEPSPARPALRAPASATSGSRKRARPPAAPGRDQARPPARRRLRLSVDEVSSPSTPEAPDIPACPSPGQKIKKSTPAAGQPPHLTSAQDQDTI
AcrIIA6+Cdt1	33560.0	MGGPKKKRKVGGGKINDDIKELILEYMSRYFKFENDFYKLPGIKFTDANWQKFKNGGTDIEKMGAARVNAMLDCLFDDFELAMIGKAQTNYYNDNSLKMNMPFYTYYDMFKKQQLLKWLKNNRDDVIGGTGRMYTASGNYIANAYLEVALESSSLGSGSYMLQMRFKDYSKGQEPIPSGRQNRLEWIENNLENIRGGSYPYDVPDYALEPSPARPALRAPASATSGSRKRARPPAAPGRDQARPPARRRLRLSVDEVSSPSTPEAPDIPACPSPGQKIKKSTPAAGQPPHLTSAQDQDTI
AcrIIA11+Cdt1	34215.4	MGGPKKKRKVGGGADMTLRQFCERYRKGDFLAKDRETQIEAGWYDWFCDDKALAGRLAKIWGILKGITSDYILDNYRVWFKNNCPMVGPLYDDVRFEPLDEEQRDELYFGVAIDDKRREKKYVIFTARNDYENECGFNNVREVRQFINGWEDELKNEEFYKAREKKRQEMEEANNKFAEIMQRADEILWNLKEDGGSYPYDVPDYALEPSPARPALRAPASATSGSRKRARPPAAPGRDQARPPARRRLRLSVDEVSSPSTPEAPDIPACPSPGQKIKKSTPAAGQPPHLTSAQDQDTI
AcrIIA13+Cdt1	27604.7	MGGPKKKRKVGGGMEVMNKSIEIKDQNNIVLIDSLGQFFTDIENDNNGRYNIDYVLLNEVEHDNGNTYYEVGMYRTEEVPFSDKVTQDNVELLEDKWLQIDQQGESYVESIFFENEEDAREYIKLVLKGHETFEETAKAIGVIKGGSYPYDVPDYALEPSPARPALRAPASATSGSRKRARPPAAPGRDQARPPARRRLRLSVDEVSSPSTPEAPDIPACPSPGQKIKKSTPAAGQPPHLTSAQDQDTI
AcrIIA14+Cdt1	24168.4	MGGPKKKRKVGGGMKSVKYISNMSKQEKGYRVYVNVVNEDTDKGFLFPSVPKEVIENDKIDELFNFEHHKPYVQKAKSRYDKNGIGYKIVQLDEGFQKFIELNKEKMKENLDYGGSYPYDVPDYALEPSPARPALRAPASATSGSRKRARPPAAPGRDQARPPARRRLRLSVDEVSSPSTPEAPDIPACPSPGQKIKKSTPAAGQPPHLTSAQDQDTI
AcrIIA15+Cdt1	25638.0	MGGPKKKRKVGGGVDTWIVYRGRTADMNKSYIAEGSTYEEVYNNFVDKYGYDVLDEDIYEIQLLKKNGENLDDYDVDSDGINNYDKLDEFRESDYVDLEDYDYRELFENSSSQVYYHEFEITHEGGSYPYDVPDYALEPSPARPALRAPASATSGSRKRARPPAAPGRDQARPPARRRLRLSVDEVSSPSTPEAPDIPACPSPGQKIKKSTPAAGQPPHLTSAQDQDTI
AcrIIA16+Cdt1	35771.5	MGGPKKKRKVGGGMGYIGTKRSERSQDAIEDYEVPLNHFNKDLIQAFIDENEAYDTLKTKKVRLWKFVAPRAGATSWHHTGTYYNKTDHYSLEKVADELLQNGDEWEEQFKAYVKEEQETATSEPVFLSVIKVQIWGGSMKRPKLVGHEVVMGVKKEGWLHAVSKATQSKYKLSANKVEMQKHYSLEDYSALTKDFPEFKAQKRAINKKMKEMYNGGSYPYDVPDYALEPSPARPALRAPASATSGSRKRARPPAAPGRDQARPPARRRLRLSVDEVSSPSTPEAPDIPACPSPGQKIKKSTPAAGQPPHLTSAQDQDTI
AcrIIA21+Cdt1	25046.5	MGGPKKKRKVGGGMDYDNENYLIPKILLQDDFYSSLSAKDILVYAVLKDRQIEALEKGWIDTDGSIYLNFKLIELAKMFSCSRTTMIDVMQRLEEVNLIERERVDVFYGYSLPYKTYINEVGGSYPYDVPDYALEPSPARPALRAPASATSGSRKRARPPAAPGRDQARPPARRRLRLSVDEVSSPSTPEAPDIPACPSPGQKIKKSTPAAGQPPHLTSAQDQDTI
AcrIIC1+Cdt1	22826.9	MGGPKKKRKVGGGMKEVFKLKPELVTYKGCGWALACIKDGEIIDLTYVRDLGIEEYDENFDGLEPEIIYYDVVASQACKEVAYRYEEMGEFTFGLCSCWEFNVMGGSYPYDVPDYALEPSPARPALRAPASATSGSRKRARPPAAPGRDQARPPARRRLRLSVDEVSSPSTPEAPDIPACPSPGQKIKKSTPAAGQPPHLTSAQDQDTI

Tables S3 The data used to generate the graphs in the corresponding figures.

Figure 2B												
% of indels	SauCas9	SauCas9/gRNA	AcrIIA4	AcrIIA5	AcrIIA6	AcrIIA11	AcrIIA13	AcrIIA14	AcrIIA15	AcrIIA16	AcrIIA21	AcrIIC1
	3.7	61.5	71.1	2.4	64.2	62.3	1.5	2.2	7.5	42.6	81.1	14.9
	1.5	73.4	77.3	2.1	73.1	70.7	0	1.4	5.8	69.3	86.9	15.1
	7.5	77.6	76	4.3	46.6	67.9	1.5	5.1	8.3	84.3	71.9	35.3
	0.3	57.2	20	1.5	51.3	85.2	3.2	3.8	8.8	59	75	28.9
	6.1	57.2	35.2	0	11.7	65.4	2.3	4	8.1	70.8		25.5
	2	66.7	80.9	1.6	79	32						
	1.5	44.8	67.7	2.5	65.5	68						
	1.2			1.7		51.1						
	0.8			1.5		44.2						
	1.7			3.4		87.1						
	0.6			2.8		77.1						
	1.9			1.8		59.2						
Average	2.4	62.6	61.2	2.1	55.9	64.2	1.7	3.3	7.7	65.2	78.7	23.9
Standard Deviation	2.2	11.1	23.7	1.1	22.6	16.1	1.2	1.5	1.2	15.5	6.7	8.9
p-value compared with SauCas9/gRNA	N/A		0.943	<0.0001	0.363	0.242	<0.0001	<0.0001	<0.0001	0.0317	0.996	0.0426

Figure 2C												
% of indels	SauCas9	SauCas9/gRNA	AcrIIA4+Cdt1	AcrIIA5+Cdt1	AcrIIA6+Cdt1	AcrIIA11+Cdt1	AcrIIA13+Cdt1	AcrIIA14+Cdt1	AcrIIA15+Cdt1	AcrIIA16+Cdt1	AcrIIA21+Cdt1	AcrIIC1+Cdt1
	3	84.3	83.5	3.2	60.9	27.3	40.1	23.2	32.3	65	73.4	57
	1.2	77.3	80.9	11	67.5	42.8	10.6	5.4	10.2	46.6	70.5	45.5
	1.8	36.6	77.8	0.6	41.9	53.7	0.2	0	0	35.8	61.7	44.7
	3.7	72	49.4	34.9	39	69.5	7.7	3.7	4.3	42.8	76.1	46.8
	1.6	60.1	76.1	3.2	50.5	70.1	12.4	8.5	17.2	49.4	85.2	53
	9.8	83.4	85.2	3.9	80.6	60.7	12.5	5	6.4	58	74.8	54.9
	11.2			12		19						
	3.6			3.9		73.1						
	4.4			0.2		19.3						
	0.8			4.5		84						
	5			7.9		83.7						
	1.7			2.7		80.1						
	2.7			4.5		61						
Average	3.9	69.0	75.5	7.1	56.7	57.3	13.9	7.6	11.7	49.6	73.6	50.3
Standard Deviation	3.2	18.2	13.2	9.1	16.0	23.4	13.6	8.1	11.6	10.5	7.7	5.3
p-value compared with SauCas9/gRNA	N/A		>0.999	<0.0001	0.879	>0.999	<0.0001	<0.0001	<0.0001	>0.999	0.13	<0.0001

Figure 3A						
% of indels	SauCas9	SauCas9/gRNA	1:1	1:2	1:5	1:10
	1.5	61.5	70.7	67.1	48.2	27.8
	7.5	73.4	67.9	78.8	65.5	57.3
	0.3	77.6	77.6	77.2	68.9	37.9
	6.1	57.2	65.4	54.7	57.1	30.8
Average	3.9	67.4	70.4	69.5	59.9	38.5
Standard Deviation	3.5	9.6	5.3	11.1	9.3	13.3
p-value compared with SauCas9/gRNA	NA		0.988	0.998	0.682	0.0015

Figure 3B						
% of indels	SauCas9	SauCas9/gRNA	1:1	1:2	1:5	1:10
	1.5	61.5	77.8	30.2	21.2	9.3
	1.6	77.3	70.1	45.1	5.1	11.3
	9.8	57.2	71.9	9.5	11.2	19.3
Average	4.3	65.3	73.3	28.3	12.5	13.3
Standard Deviation	4.8	10.6	4.0	17.9	8.1	5.3
p-value compared with SauCas9/gRNA	NA		0.774	0.0022	<0.0001	0.0001

Figure 4D			
HDR	Control	AcrIIA11+Cdt1	AcrIIA16+Cdt1
	8	12.1	13.8
	5.2	12.5	10.2
	4	13.4	12.3
Average	5.7	12.7	12.1
Standard Deviation	2.1	0.7	1.8
p-value compared with Control		0.0035	0.0054

Figure 4E				
HDR	1:1	1:2	1:5	1:10
	10	13.6	15.8	3.8
	11.2	12.8	13.8	2.5
	6.9	16.4	17.5	2.4
Average	9.4	14.3	15.7	2.9
Standard Deviation	2.2	1.9	1.9	0.8
p-value compared with (1:1)		0.0237	0.006	0.0053

Figure 4F			
HDR	Control	AcrIIA11+Cdt1	AcrIIA16+Cdt1
	0.3	3.9	1.6
	0.4	3	1.9
	0	2.9	1.2
	0.4	2.2	1.9
Average	0.28	3.00	1.65
Standard Deviation	0.19	0.70	0.33
p-value compared with Control		<0.0001	0.004

Supplemental Methods

Plasmid construction

pAAV_SauCas9_gRNA(EMX1): The experimental procedure was followed for the instruction manual of AAVpro® CRISPR/SaCas9 Helper Free System (AAV2) (Takara Bio). Oligo-1 (EMX1) and Oligo-2 (EMX1) were synthesized by Eurofins Genomics. The fragments were annealed and ligated to pAAV-Guide-it-1 Vector using DNA Ligation Kit＜Mighty Mix＞ (Takara Bio), resulting pAAV_SauCas9_gRNA(EMX1).

Oligo-1(EMX1); 5'-ACCGGCAACCACAAACCCACGAGGG-3'

Oligo-2(EMX1); 5'-AAACCCCTCGTGGGTTTGTGGTTGC-3'

pAAV_SauCas9_gRNA(VEGFA): The plasmid was constructed in the same procedure as pAAV_SauCas9_gRNA (EMX1) using the following Oligo-1 and Oligo-2.

Oligo-1(VEGFA); 5'-ACCGGGGTGAGTGAGTGTGTGCGTG-3'

Oligo-2(VEGFA); 5'-AAACCACGCACACACTCACTCACCC-3'

pAAV_SauCas9_gRNA(FANCF): The plasmid was constructed in the same procedure as pAAV_SauCas9_gRNA (EMX1) using the following Oligo-1 and Oligo-2.

Oligo-1(FANCF); 5'-ACCGGCAAGGCCCGGCGCACGGTGG-3'

Oligo-2(FANCF); 5'-AAACCCACCGTGCGCCGGGCCTTGC-3'

pAAV_SauCas9: pAAV_SauCas9_gRNA(EMX1) was digested with EcoRI/NotI, then blunt end treatment was performed by using Klenow Fragment (Takara Bio). The plasmid was ligated using DNA Ligation Kit<Mighty Mix> (Takara Bio), resulting pAAV_SauCas9.

pAAV_Acrs-Cdt1_gRNA (Acrs, AcrIIA4/A5/A6/A11): DNA fragments for NLS_Acrs_HA (Acrs; AcrIIA4/A5/A6/A11) were synthesized by Eurofins Genomics. Each fragment was PCR amplified with pFucci primers and digested with BamHI/XhoI. The same treatment was done for pFucci-G1 Orange Expression vector (MBL) and the fragments were ligated to the N-terminus of hCdt1(30-120) sequence by using DNA Ligation Kit<Mighty Mix> (Cat# 6023, Takara Bio) resulting pFucci_Acrs-Cdt1. Acrs-Cdt1 sequences were PCR amplified with pAAV Acr primers, and digested with AflII/BamHI. The same treatment was done for pAAV_SauCas9_gRNA, and the fragments were ligated with DNA Ligation Kit<Mighty Mix> (Takara Bio), resulting pAAV_AcrIIA4/A5/A6/A11+Cdt1_gRNA.

pFucci Fw primer; 5'-GAGCTCGGATCCGCCACCATGGGTGGCCCGA-3'

pFucci Rv primer; 5'-ATTATTCTCGAGGGCGTAGTCGGGCACGTC-3'

pAAV Acr Fw primer; 5'-GGAGAAGACTTAAGGCCGCCACCATGGGTGGCCCGAAGAAG-3'

pAAV Acr Rv primer; 5'-CCTCTTCTGGATCCTTAGATGGTGTCCTGGTCCTGCGC-3'

pAAV_Acrs-Cdt1_gRNA (Acrs; AcrIIA13/A14/A15/A16/A21/C1): DNA fragments for NLS_Acrs_HA (Acrs; AcrIIA13/A14/A15/A16/A21/C1) were synthesized by Eurofins Genomics.

Each fragments were PCR amplified by pAAV_AcrIIA13-A16/A21/C1+Cdt1_gRNA primers and the fragments were assembled with AflII/XhoI digested pAAV_AcrIIA5+Cdt1_gRNA(EMX1) using NEBuilder® HiFi DNA Assembly Master Mix (New England Biolabs), resulting pAAV_AcrIIA13/A14/A15/A16/A21/C1+Cdt1_gRNA.

pAAV_AcrIIA13-A16/A21/C1+Cdt1_gRNA Fw;

5'-AGCTGGTTTAGTGGATATCCTTAAGGC-3'

pAAV_AcrIIA13-A16/A21/C1+Cdt1_gRNA Rv;

5'-CGGGCCTGGCGGGGCTGGGCTCGAGGGCGTAGTCGGGCACGTCGT-3'

pAAV_Acrs_gRNA:

Acr genes were amplified by PCR using pAAV_Acrs-Cdt1_gRNA plasmids as templates and pAAV_Acr_gRNA primers. The fragments were assembled with AflII/BamHI digested pAAV_AcrIIA11+Cdt1_gRNA(EMX1) using NEBuilder® HiFi DNA Assembly Master Mix (New England Biolabs), resulting pAAV_Acrs_gRNA (Acrs; AcrIIA4/A5/A6/A11/ A13/A14/A15/A16/A21/C1).

pAAV_Acr_gRNA Fw;

5'-AGCTGGTTTAGTGGATATCCTTAAGGC-3'

pAAV_Acr_gRNA Rv;

5'-TGGCAACTAGAAGGCACAGGGATCCTTAGGCGTAGTCGGGCACGTCGT-3'

pAAV_Acrs-Cdt1_gRNA_HDR template (Acrs; AcrIIA5, A11, A13, A14, A15, A16, C1):

5' and 3' homology arm (HA) fragments were amplified by PCR with EMX1 5' HA primers and EMX1 3' HA primers, respectively, using genomic DNA extracted from 293A cells. The amplified HA fragments were assembled using NEBuilder® HiFi DNA Assembly Master Mix (New England Biolabs), resulting the HDR template fragment. The fragment was inserted to NotI digested pAAV_Acrs-Cdt1_gRNA using genomic DNA extracted from 293A cells. The amplified HA fragments were assembled using NEBuilder® HiFi DNA Assembly Master Mix (New England Biolabs), resulting pAAV_Acrs-Cdt1_gRNA(EMX1)_HDR template.

EMX1 5'-HA Fw; 5'-ACTTGTTGGCGAGATTTTTGCCTCTTGATATAAACACCACCTC-3'

EMX1 5'-HA Rv; 5'-CACCCTAGAATTCGTCATTGGAGGTGACATCGA-3'

EMX1 3'-HA Fw; 5'-CAATGACGAATTCTAGGGTGGGCAACCACAAAC-3'

EMX1 3'-HA Rv; 5'-CTAGGGGTTCCTAGATCTGCTCTAGGCAGAGGGAAGCACT-3'

pAAV_gRNA_HDR template:

pAAV_AcrIIA5+Cdt1_gRNA_HDR template was digested with AflII/EcoRI to remove AcrIIA5+Cdt1 gene and the polyA sequence. Oligonucleotides including T7 &T3 promoter sequences were synthesized by Eurofins Genomics. The oligonucleotides were annealed and resulting double-stranded fragment was ligated to digested pAAV_AcrIIA5+Cdt1_gRNA_HDR template using DNA Ligation mix<Mighty Mix> (Takara Bio), resulting pAAV_gRNA_HDR template.

pAAV_T7&T3_1;
5'-TTAAGTAATACGACTCACTATAGGGGCAATTAACCCTCACTAAAGGGGTACCG-3'
pAAV_T7&T3_2;
5'-AATTCGGTACCCCTTTAGTGAGGGTTAATTGCCCCTATAGTGAGTCGTATTAC-3'

Western blotting
Proteins within each sample were separated by SDS-PAGE using either the NuPAGE electrophoresis system (Thermo Fisher) (Figure S1A and S1B) or the standard Laemmli's method (Figure S1C), then transferred to nitrocellulose membranes. The membranes were blocked with 4% skim milk in TBS-T (0.1% Tween 20 in Tris-buffered saline) for 30 min, followed by incubation with the primary antibody. After washing with TBS-T, the membranes were incubated with the appropriate horseradish peroxidase-conjugated secondary antibody, and immunoreactivity was detected using ECL detection reagents. For the detection of SauCas9 and Acrs, mouse monoclonal anti-FLAG (A8592, Sigma-Aldrich) and rat monoclonal anti-HA (12158167001, Sigma-Aldrich) were used as primary antibodies, respectively. Anti-rat IgG HRP-linked antibody (ab97057, Abcam) and anti-rabbit IgG HRP-linked antibody (#7074, Cell Signaling Technology) were used as secondary antibodies. Polyclonal anti-α-tubulin (PM054, MBL Life Science) was used as a loading control.

T7E1 assay
293A cells were seeded in 12-well plates at a density of 1.0×10^5 cells/well. Twenty-four hours post-seeding, the cells were infected with AAV2(SauCas9/gRNA) at MOIs of 5.0×10^2, 1.0×10^3, 5.0×10^3, 1.0×10^4, 5.0×10^4, and 1.0×10^5, except for the FANCF site (5.0×10^2, 1.0×10^3, 5.0×10^3, 1.0×10^4). The titers of AAV2 were 5.4×10^8 gc/μL for AAV2(SauCas9/gRNA(EMX1)), 5.3×10^8 gc/μL for AAV2(SauCas9/gRNA(VEGFA)), and 1.0×10^8 gc/μL for AAV2(SauCas9/gRNA(FANCF)). The viral titer was adjusted to 1.0×10^8 gc/μL with TNE buffer and the calculated specific viral load was applied directly. Forty-two hours post-infection, half of the proliferated cells were passaged into a new 12-well plate. Genomic DNA was extracted 24 hours later using the QIAamp DNA Mini Kit (QIAGEN). The target regions were PCR-amplified using Tks Gflex™ DNA Polymerase (Takara Bio), followed by purification using the MinElute PCR Purification Kit (Qiagen).
The following primer pairs were used for PCR;
EMX1;
Fw (5'-GGAGCAGCTGGTCAGAGGGG-3') and Rv (5'-GGGAAGGGGGACACTGGGGA-3')
VEGFA;
Fw (5'-AGATGGCACATTGTCAGAGG-3') and Rv (5'-GAAACTCTGTCCAGAGACACG-3')
FANCF;
Fw (5'-GGGCCGGGAAAGAGTTGCTG-3') and Rv (5'-GCCCTACATCTGCTCTCCCTCC-3')

For the T7E1 assay, 200 ng of the amplified DNA fragment and 1 μL of 10× NEBuffer 2 (New England Biolabs) were mixed with H_2O to a total volume of 9 μL. Re-annealing was performed by heating at 95°C for 5 min, cooling to 85°C at -2°C/s, and further cooling to 25°C at -0.1°C/s, followed by holding at 4°C. After annealing, 1 μL of T7 Endonuclease I (New England Biolabs) was added, and the mixture was incubated at 37°C for 1 hour. The resulting DNA fragments were analyzed via electrophoresis.
