# Supplementary material for: SauCas9-based cell cycle-dependent genome editing via AAV delivery
Source: Mol Ther Adv. 2026 May 6;34(2):201751. doi: 10.1016/j.omta.2026.201751 (PMC13213762; doi:10.1016/j.omta.2026.201751)
Supplement: Document S2. Article plus supplemental information [file mmc2.pdf]

Molecular Therapy
Advances
Original Article

SauCas9-based cell cycle-dependent genome editing via AAV delivery

Erina Matsugi,[1] Kanae Kishi,[1] Ayane Kishi,[1] Kohei Nagase,[2] Kiyomi Nigorikawa,[1,2] and Wataru Nomura[1,2]

[1]Graduate School of Biomedical and Health Sciences, Hiroshima University, 1-2-3 Kasumi, Minami-ku, Hiroshima 734-8553, Japan; [2]School of Pharmaceutical Sciences, Hiroshima University, 1-2-3 Kasumi, Minami-ku, Hiroshima 734-8553, Japan

The CRISPR-Cas system is a widely used genome editing technology with diverse applications. Although homology-directed repair (HDR) offers precise gene editing, its efficiency is typically lower than that of non-homologous end joining (NHEJ). Building on our previous cell cycle-dependent genome editing system for *Streptococcus pyogenes* Cas9 (SpyCas9), we adopted this approach for *Staphylococcus aureus* Cas9 (SauCas9) to enable efficient adeno-associated virus (AAV) delivery. To enhance HDR efficiency and editing accuracy in the context of AAV delivery, we developed a cell cycle-dependent genome editing system. We screened 10 anti-CRISPR (Acr) candidates and identified AcrIIA5, A13, A14, A15, and C1 as potent inhibitors of SauCas9. The fusion of these Acrs with the Cdt1(30–120) fragment restricted SauCas9 activity to the S/G2 phases, where HDR is predominant. Although AcrIIA11 and AcrIIA16 alone showed weak inhibition, their Cdt1 fusions (AcrIIA11+Cdt1 and AcrIIA16+Cdt1) showed a 2-fold increase in HDR efficiency within the AAV delivery system. This AAV-based, cell cycle-dependent SauCas9 system, which leverages optimized Acr-Cdt1 fusions, holds promise for improving the efficiency and accuracy of *in vivo* genome editing. Its small size is ideal for AAV packaging and may offer fewer off-target effects.

INTRODUCTION

The CRISPR-Cas system is a widely used genome editing technology with applications in gene therapy, plant science, animal breeding, and other fields. In the CRISPR-Cas system, double-strand breaks induced by the Cas9 nuclease complexed with guide RNA (gRNA) at the target site are repaired via two main cellular pathways: non-homologous end joining (NHEJ) and homology-directed repair (HDR). The HDR pathway accurately repairs the target sequences using a donor template, making it an ideal mechanism when high fidelity is required. However, HDR is less efficient than NHEJ, and improving HDR efficiency is a key goal for achieving safer and more accurate genome editing.

HDR is predominantly active during the S and G2 phases of the cell cycle. To leverage this, cell cycle-dependent genome editing was developed fusing anti-CRISPR (Acr) proteins to the N-terminal region of chromatin licensing and DNA replication factor 1 (Cdt1). For *Streptococcus pyogenes* Cas9 (SpyCas9), AcrIIA4 is a potent inhibitor. The expression of an AcrIIA4+Cdt1(30–120) fusion protein, together with SpyCas9, increases HDR efficiency and suppresses off-target effects.[1] Cdt1 accumulates in the nucleus during the G1 phase and is subsequently degraded during the S and G2 phases by the SCF^{Skp2} E3 ligase complex. This cell cycle-dependent recognition by E3 ligases is specifically limited to the SCF^{Skp2} pathway when the Cdt1 domain is shortened to residues 30–120. This truncated domain retains the primary ubiquitylation signals targeted by the SCF^{Skp2} complex, which recognizes the degron when it is phosphorylated at Ser31 and/or Thr29 during the S/G2 phases.[2,3] Consequently, the AcrIIA4+Cdt1 fusion protein inhibits SpyCas9 during G1 and is degraded during S/G2, thereby activating SpyCas9 when HDR is most active. This inhibition/activation cycle is repeated with cell cycle progression,[1,4–6] a mechanism indirectly supported by a recent study using the CRISPR activation (CRISPRa) system.[7]

Because this system is autonomously regulated by the cell cycle without external stimuli such as light or chemicals, it is potentially compatible with *in vivo* genome editing. Acr proteins are natural CRISPR-Cas antagonists encoded by diverse mobile genetic elements, such as plasmids and phages, which inhibit CRISPR-Cas function through diverse mechanisms.[8–12] Numerous Acrs have been identified across various CRISPR-Cas systems,[13–20] suggesting that cell cycle-dependent genome editing could be adapted to other Cas nucleases by selecting appropriate Acrs.[21]

For *in vivo* applications, compatibility with adeno-associated virus (AAV) delivery is crucial. AAV is widely used because of its low immunogenicity and high transduction efficiency, but its packaging capacity is limited to approximately 4.7 kilobases (kb). The SpyCas9 gene (∼4.2 kb) exceeds this limit when combined with regulatory elements and gRNA cassettes, often requiring dual-AAV vector strategies.[22–24] In such a system, the SpyCas9 gene is split between two vectors (a 5′ vector with promoter, 5′ SpyCas9 fragment, and donor splice signal and a 3′ vector with acceptor splice signal, 3′ SpyCas9 fragment, and poly(A) signal). Co-transduction of vectors carrying

Received 15 September 2025; accepted 1 May 2026;
https://doi.org/10.1016/j.omta.2026.201751.

Correspondence: Wataru Nomura, Graduate School of Biomedical and Health Sciences, Hiroshima University, 1-2-3 Kasumi, Minami-ku, Hiroshima 734-8553, Japan.
E-mail: wnomura@hiroshima-u.ac.jp

© 2026 The Authors.
Published by Elsevier Inc. on behalf of The American Society of Gene and Cell Therapy.
This is an open access article under the CC BY license (http://creativecommons.org/licenses/by/4.0/).

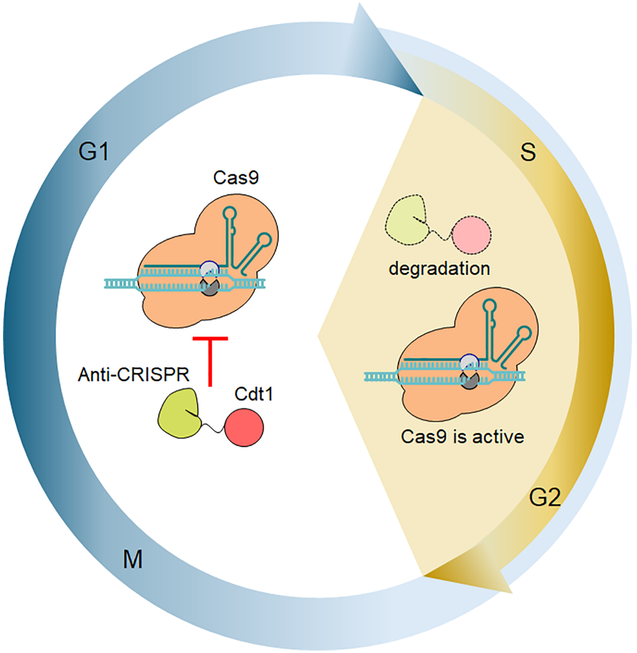

Figure 1. Overview of cell cycle-dependent genome editing using Acr fusion with Cdt1

Cdt1 is expressed and accumulates in the nucleus during the G1 phase and is degraded in the S/G2 phases. The fusion protein of Acr and Cdt1 can suppress CRISPR-Cas9 genome editing during the G1 phase, and CRISPR-Cas9 is reactivated in the S/G2 phases.

split SpyCas9 fragments allows reconstitution of the full-length transcript via concatemerization and splicing; however, this process is inefficient.

Therefore, smaller Cas9 orthologs are required to overcome this limitation. SauCas9, derived from *S. aureus*, is a small gene (approximately 3.2 kb), making it ideal for packaging into a single AAV vector. In this study, we developed a SauCas9-based cell cycle-dependent genome editing system that utilizes AAV delivery (Figure 1). We screened 10 Acr candidates for optimal SauCas9 inhibition and created Cdt1 fusions for promising candidates to evaluate their potential to increase HDR efficiency and accuracy.

RESULTS

We initially explored the optimal Acr proteins for SauCas9 by testing the inhibitory activity of 10 candidates selected based on availability and prior reports, including Acrs whose inhibitory activity against SauCas9 had not been reported (Table 1). Two AAV vectors were prepared: pAAV_SauCas9 for SauCas9 expression and pAAV_Acr/Acr-Cdt1_gRNA for Acr or Acr-Cdt1 expression and gRNA transcription to ensure the presence of Acr or Acr-Cdt1 in cells with an active SauCas9/gRNA complex for inhibition assessment (Figure 2A). An AAV vector containing the SauCas9 and a gRNA expression cassette (pAAV_SauCas9/gRNA) served as a positive control. AAV serotype 2, with broad tissue tropism, was used in this study. Three candidate sequences (EMX1, VEGFA, and FANCF)[29] were tested for editing, using a positive control (Figure S1). High editing efficiency was observed for each target using T7E1 assays, and EMX1 was selected for this study because it had the fewest off-target sites. The prepared AAV2 was used to infect 293A cells at a multiplicity of infection (MOI) of 1.0×10^4. At the EMX1 site, editing efficiency saturation was observed at MOIs exceeding 5.0×10^4. Consequently, the MOI was set at 1.0×10^4 to ensure that excessive SauCas9 activity did not compromise the evaluation of Acr candidates. The expression of SauCas9, Acr, and Acr-Cdt1 fusions was evaluated under the same conditions as those used in the genome editing assays. Western blotting results indicated that Acr expression levels were lower than those of the Acr-Cdt1 fusion proteins (Figures S2A and S2B). Acr expression was also assessed in the presence of the proteasome inhibitor MG132 and showed increased expression levels after MG132 treatment. These results suggested that the Acrs were rapidly degraded in cells and that

Table 1. Inhibitory mechanisms for Acr candidates

Acr	Reported inhibitory mechanism	Representative Cas9 targeted for inhibition	Reference
AcrIIA4	By preventing HNH domain movement toward the cleavage site	LmoCas9/SpyCas9	Dong et al.[8]
AcrIIA5	By inhibiting the activity of the RuvC nuclease domain	SpyCas9/SauCas9/St1Cas9/CjeCas9	Song et al.[11]
AcrIIA6	By preventing the DNA binding of Cas9	St1Cas9	Fuchsbauer et al.[25]
AcrIIA11	By inhibiting DNA cleavage	SpyCas9/SauCas9	Dillard et al.[26]
AcrIIA13	By preventing target DNA loading	SauCas9	Watters et al.[20]
AcrIIA14	By preventing the DNA binding of Cas9	SauCas9	Liu et al.[27]
AcrIIA15	By blocking PAM recognition	SpyCas9/SauCas9	Deng et al.[28]
AcrIIA16	By inhibiting gRNA loading	SpyCas9/SauCas9/St1Cas9/CjeCas9	Mahendra et al.[17]
AcrIIA21	Unknown	SpyCas9/SauCas9/SinCas9	Eitzinger et al.[13,a]
AcrIIC1	By inhibiting DNA cleavage	SauCas9/NmeCas9/CjeCas9	Mahendra et al.[17]

[a]Inhibitory activity has been reported, but no inhibitory mechanisms.

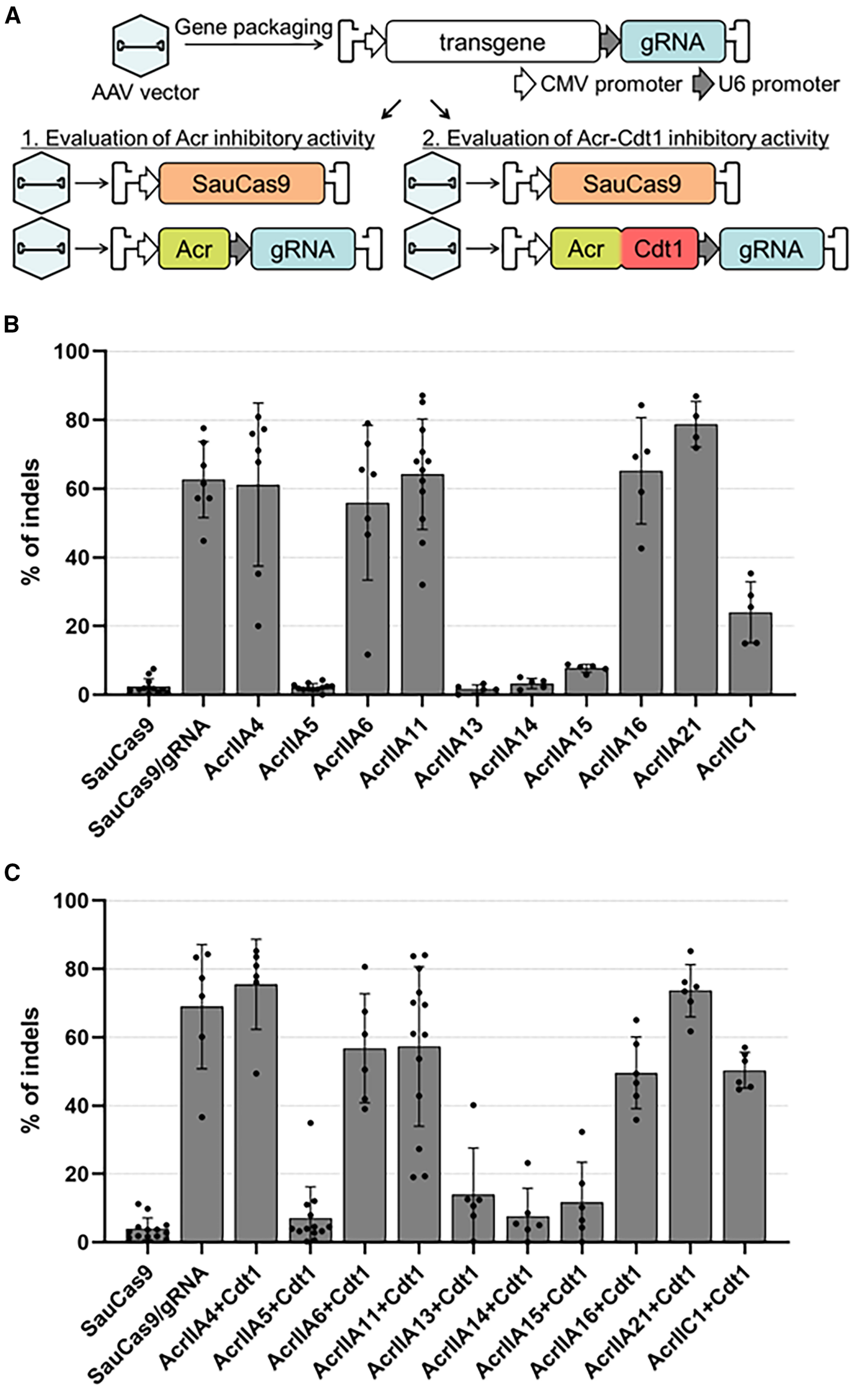

Figure 2. Design of AAV vectors and screening of Acr candidates for SauCas9 inhibition

(A) Schematic diagram of AAV vector constructs. The diagram illustrates the design of vectors used to package SauCas9, Acr/gRNA, and Acr-Cdt1/gRNA expression cassettes. The gRNA expression cassette was included in the sequences expressing Acr or Acr-Cdt1 fusions. (B and C) Screening of 13 Acr candidates and their respective Cdt1 fusions for inhibitory activity against SauCas9. (B) Inhibitory activity of Acr candidates on SauCas9-mediated genome editing. The SauCas9 control represents cells transduced with AAV2(SauCas9) only. The SauCas9/gRNA control represents cells transduced with AAV2(SauCas9/gRNA). (C) Inhibitory activity of Acr-Cdt1 fusions on SauCas9-mediated genome editing. Control conditions are identical to those described in (B). Statistical significance was analyzed by one-way ANOVA followed by Dunnett's post hoc test. Calculated *p* values are summarized in Table S3.

the integrity of the pAAV expression constructs was maintained (Figure S2C).

Inhibitory activity was evaluated by measuring indel mutation efficiency at the EMX1 target site[30,31] using tracking of indels by decomposition (TIDE) analysis of Sanger sequencing data.[32] AcrIIA5, A13, A14, A15, and C1 were confirmed as potent SauCas9 inhibitors as previously reported, and no new potent inhibitors were identified (Figure 2B). Next, we investigated the inhibitory activity of the Cdt1(30–120) fusions against all the Acr candidates. AcrIIA5, A13, A14, and A15 showed inhibitory activity in the form of a Cdt1 fusion, comparable to that of the unfused Acrs (Figure 2C). The inhibitory activity of AcrIIC1+Cdt1 was attenuated compared with that of AcrIIC1 alone. Western blotting indicated increased stability of most Cdt1-fused Acr proteins compared to the unfused proteins, except for AcrIIA4. Additionally, AcrIIA16+Cdt1 exhibited weak inhibitory activity, whereas AcrIIA16 alone did not. This enhancement may stem from the increased nuclear localization of Cdt1-fused Acrs during G1, compared with Acr with only a standard nuclear localization signal. For AcrIIA11, an increased amount of AcrIIA11 enhances inhibition activity against SpyCas9, and dimer formation of AcrIIA11 is observed in size exclusion chromatography.[14] As the apparent inhibitory activity of AcrIIA11 was not shown when AAV2 for AcrIIA11 expression was added in the ratio of 1:1 with AAV2 for SauCas9 expression (AAV2(SauCas9)), the effect of increased AcrIIA11 levels was investigated.[14] Inhibitory activity increased proportionally with the amount of AcrIIA11 or AcrIIA11+Cdt1, particularly for the Cdt1-fusion, suggesting that AcrIIA11 dimer formation may also be important for SauCas9 inhibition (Figures 3A and 3B). Based on these results, AcrIIA5, A13, A14,

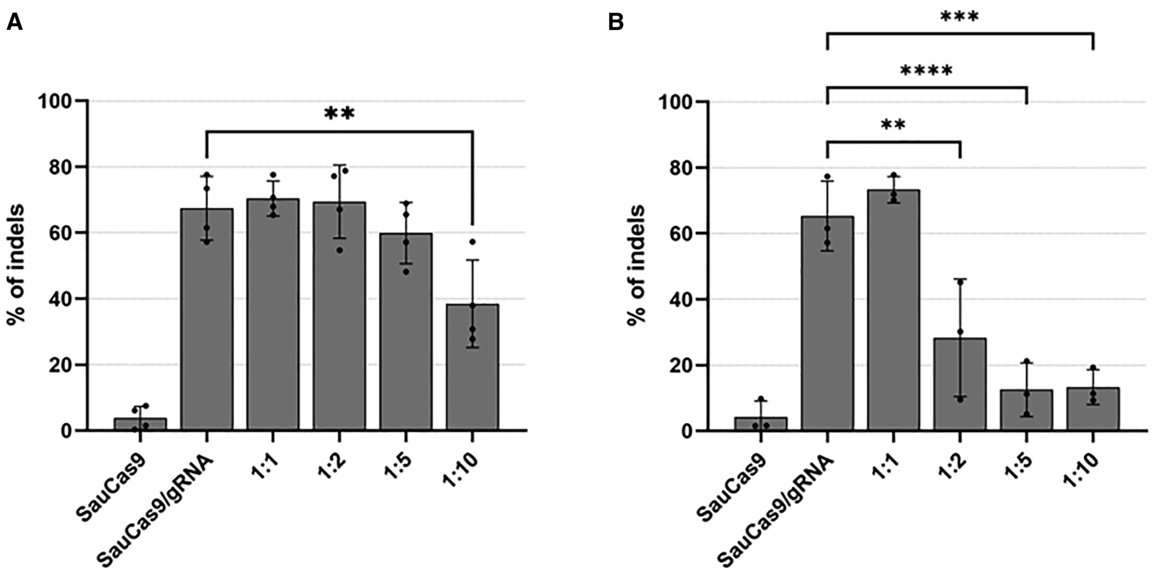

Figure 3. Analysis of dose-dependent SauCas9 inhibition by AcrIIA11 variants

The potency of AcrIIA11 (A) and the fusion protein AcrIIA11+Cdt1 (B) in inhibiting SauCas9 was evaluated through a dose-response assay. The MOI of AAV2(SauCas9) was held constant at 1.0×10^4, whereas the MOI of AAV2(AcrIIA11/gRNA) or AAV2(AcrIIA11+Cdt1/gRNA) was increased as indicated: 1:1 (1.0×10^4), 1:2 (2.0×10^4), 1:5 (5.0×10^4), and 1:10 (1.0×10^5). The rate of indel mutations was evaluated using the TIDE program. Statistical significance was analyzed by one-way ANOVA followed by Dunnett's post hoc test (**$p < 0.005$, ***$p < 0.0005$, ****$p < 0.0001$). Calculated p values are summarized in Table S3.

and A15 were selected as strong inhibitors for testing in cell cycle-dependent genome editing. In addition, AcrIIA16 and AcrIIC1 were selected as moderate inhibitors, as they exhibited indel efficiencies of less than 50%. AcrIIA11 was also included as a candidate due to its dose-dependent inhibitory activity against SauCas9.

The AAV single-stranded genome can serve as a donor template for HDR.[33,34] We inserted an HDR template sequence containing an EcoRI site flanked by 730 bases with homology arms targeting the *EMX1* site into AAV2(Acrs-Cdt1/gRNA) (Figures 4A and 4B). SauCas9 genome editing was performed using AAV2s encoding Cdt1-fused Acr candidates selected in primary screening, along with an integrated gRNA cassette and HDR template. HDR efficiency was assessed by EcoRI digestion of the EMX1 PCR amplicon on a MultiNA microchip electrophoresis. Of these candidates, only AcrIIA11+Cdt1 and AcrIIA16+Cdt1 yielded detectable EcoRI-digested fragments (Figure 4C). It is plausible that stronger inhibitors (AcrIIA5, A13, A14, A15) significantly suppress HDR, even when fused to Cdt1. The HDR efficiencies calculated by the tracking of insertions, deletions and recombination events (TIDER) program were approximately 2-fold higher than those of the control using AAV2(gRNA/HDR template). This statistically significant difference indicates successful regulation of Acr-Cdt1 inhibition during the G1 phase, effectively biasing the repair outcomes toward the HDR pathway (Figure 4D). In contrast, no apparent inhibition of indel efficiency was observed with AcrIIA11+Cdt1 or AcrIIA16+Cdt1 in the same samples (Figure S3). Off-target effects were also evaluated under HDR editing conditions at the off-target site that exhibited the highest mutation rate.[29] Although no significant change was observed in the presence of AcrIIA11+Cdt1 or AcrIIA16+Cdt1, the inherently low frequency of off-target activity at this previously identified off-target site for EMX1 may have made it difficult to detect a further reduction in editing (Figure S4).

As a proportional increase in inhibition by AcrIIA11 and AcrIIA11+Cdt1 was observed (Figure 3C), the effects of changing the MOI ratio of AAV2(AcrIIA11+Cdt1/gRNA) (1:2, 1:5, and 1:10) relative to a fixed MOI for AAV2(SauCas9) were addressed. HDR efficiency increased by 1.5- and 1.6-fold when AAV2 (AcrIIA11+Cdt1/gRNA/HDR template) was used at ratios of 1:2 and 1:5, respectively, compared with the 1:1 sample. However, when the ratio of AAV2(AcrIIA11+Cdt1/gRNA/HDR template) to AAV2(SauCas9) was 1:10, HDR efficiency decreased by 70% (Figure 4E). A successful increase in HDR efficiency was observed when AAV2(AcrIIA11+Cdt1/gRNA/HDR template) was used at ratios of 1:2 and 1:5; however, the HDR template was also increased. To determine whether the observed enhancement was influenced by the increased concentration of the donor template, we performed a control titration using an AAV2 vector carrying only the gRNA and HDR template (Figure S5). Increasing the ratio of this control vector up to 5-fold did not yield a statistically significant increase in baseline HDR efficiency, confirming that improved editing depends on the presence of the Acr-Cdt1 fusion. The indel efficiencies determined for the samples in Figure 4E are shown in Figure S6. The decrease in indel efficiency for ratios of 1:2 and 1:5 was not statistically significant.

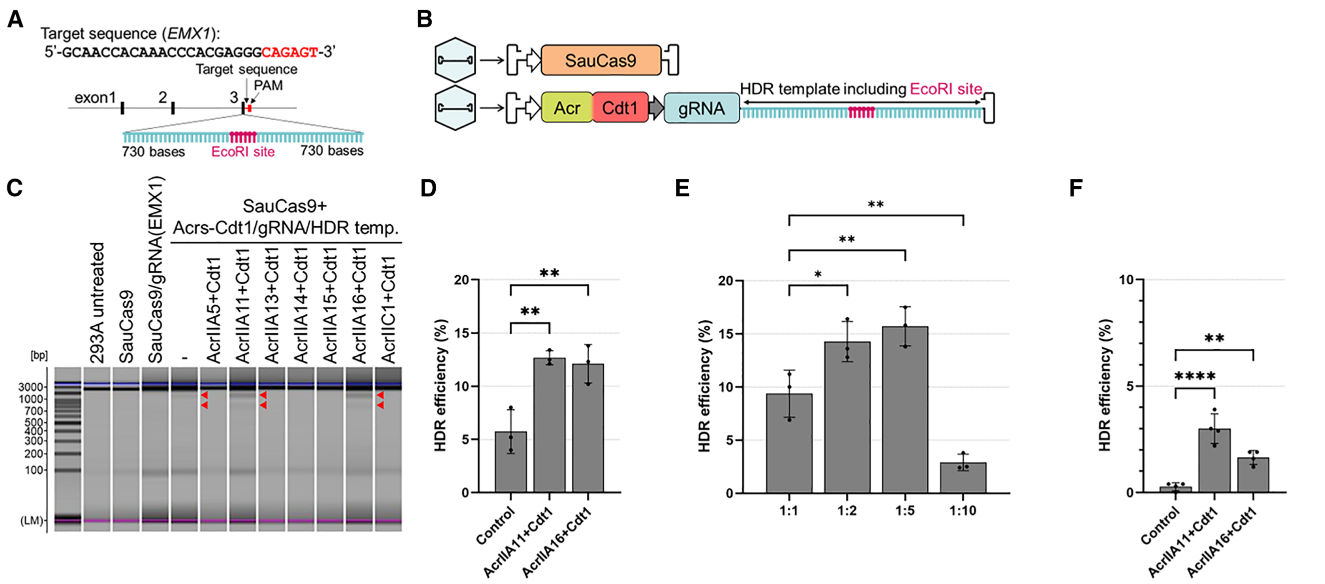

Figure 4. HDR mediated by an AAV-delivered repair template in SauCas9-based cell cycle-dependent genome editing

(A) Design of the gRNA and HDR template. The template was designed to insert an EcoRI restriction site into exon 3 of the EMX1 gene. The gRNA was designed to target the same genomic locus. (B) Schematic diagrams of AAV vectors for the expression of SauCas9 and Acr-Cdt1/gRNA/HDR template. (C) Evaluation of HDR efficiency by restriction enzyme digestion. Representative results from a MultiNA microchip electrophoresis system (Shimadzu) show fragments following EcoRI digestion. Red arrows indicate fragments that confirm successful HDR template knockin. (D) Quantification of HDR efficiency in 293A cells. The control group represents cells co-transduced with AAV2(SauCas9) and AAV2(gRNA/HDR template). The AcrIIA11+Cdt1 and AcrIIA16+Cdt1 groups represent cells co-transduced with AAV2(SauCas9) and either AAV2 (AcrIA11+Cdt1/gRNA/HDR template) or AAV2(AcrIIA16+Cdt1/gRNA/HDR template), respectively. Genomic DNA was analyzed by Sanger sequencing, and efficiencies were calculated using the TIDER program. (E) Dose-dependent effects of AcrIIA11+Cdt1 on HDR. The MOI of AAV2(SauCas9) was held constant at 1.0×10^4, whereas the MOI of AAV2(AcrIIA11+Cdt1/gRNA/HDR template) was increased as indicated: 1:1 (1.0×10^4), 1:2 (2.0×10^4), 1:5 (5.0×10^4), and 1:10 (1.0×10^5). (F) Quantification of HDR efficiency in HeLa cells. Sample descriptions are identical to those provided in (D). Statistical significance was analyzed by one-way ANOVA followed by Dunnett's post hoc test (*$p < 0.05$, **$p < 0.005$, ****$p < 0.0001$). Calculated p values are summarized in Table S3.

To evaluate the generalizability of our system across different cellular contexts, we expanded testing to include HeLa cells. Consistent with our findings in 293A cells, we observed 4.7-fold and 2.5-fold increases in HDR efficiency for AcrIIA11+Cdt1 and AcrIIA16+Cdt1 co-transduction cells, respectively. These results indicated that the Acr-Cdt1 regulatory mechanism is applicable across multiple human cell lines (Figure 4F).

DISCUSSION

Compared to SpyCas9, SauCas9 recognizes a longer PAM sequence (NNGRRT, where R is A or G), which generally correlates with lower off-target activity. This inherent specificity can synergize with cell cycle-dependent control to minimize unwanted modifications. The observed increase in HDR efficiency with AcrIIA11+Cdt1 and AcrIIA16+Cdt1 warrants further investigation of the underlying mechanisms, potentially involving the analysis of Cas9 activity dynamics and DNA repair factor recruitment across cell cycle phases. Interestingly, the strong inhibitors (AcrIIA5, A13, A14, and A15) were ineffective for cell cycle-dependent genome editing. Although the reason for this remains unclear, the balance between potent inhibition and timely release is likely a key factor in the system's success. Notably, AcrIIC1 did not show successful HDR increase despite possessing moderate inhibitory activity similar to that of AcrIIA16. As information regarding the specific inhibitory mechanisms for the Acrs used in this study remains limited (Table 1), further mechanistic studies will be essential to identify which Acr properties are most suitable for cell cycle-dependent genome editing. Western blotting analysis revealed that some Acr proteins exhibited low steady-state expression levels. Our experiments with the proteasome inhibitor MG132 showed stabilization of these proteins, suggesting that certain Acrs are inherently susceptible to proteasomal degradation. Interestingly, Cdt1 fusion appears to stabilize these Acr proteins, potentially by altering their protein turnover and reducing their susceptibility to degradation. This stabilizing effect, combined with cell cycle-specific expression, likely contributes to the efficacy of the Acr-Cdt1 system. The observation that HDR was suppressed when the AcrIIA11+Cdt1-expressing AAV was increased to a 1:10 ratio further supports the importance of maintaining an optimal stoichiometric balance.

We recently reported a CRISPRa system confirming that repeated SpyCas9 activation is cell cycle-dependent and mediated by the degradation of AcrIIA4+Cdt1 during the S/G2 phases.[7] The CRISPRa system may provide an efficient strategy for identifying the suitable Acr candidates by enabling non-invasive, real-time monitoring of Cas9 "on/off" kinetics via fluorescent reporters. This approach enables rapid identification of inhibitors with optimal

balance of potency and release, which can then be used to broaden the scope of cell cycle-dependent genome editing across a wider variety of Cas enzymes. Furthermore, the consistent enhancement of HDR observed in both 293A and HeLa cells (Figure 4F) suggested that the Acr-Cdt1 regulatory mechanism was robust across different human cellular contexts.

Our results indicate that while AcrIIA11+Cdt1 and AcrIIA16+Cdt1 fusions significantly enhanced HDR efficiency, they did not concurrently reduce the overall rate of NHEJ-mediated indels (Figure S3). This suggests that while our system successfully expands the window for HDR by restricting Cas9 activity during G1, it does not decisively shift the repair balance away from NHEJ once Cas9 is activated in S/G2. We acknowledge that the persistence of NHEJ editing may limit the immediate therapeutic enthusiasm for this system. Future optimization will be necessary to achieve a more robust suppression of error-prone repair.

For practical *in vivo* applications, optimizing AAV delivery system remains crucial, including exploring tissue-specific promoters and enhancing transduction efficiency while minimizing potential immunogenicity. The principle of cell cycle-dependent genome editing using Acr-Cdt1 fusions could be extended to other CRISPR-Cas systems beyond SauCas9. Although the current study demonstrated robust HDR enhancement *in vitro*, translating this system *in vivo* remains a critical future direction. We recognize two primary hurdles for clinical translation. First, while this study provides a mechanistic proof of concept using a small EcoRI site insertion, we have not yet evaluated the system's ability to facilitate the integration of larger therapeutic transgenes. The integration efficiency of larger cargos, which often face greater steric and cellular hurdles, remains a critical area for future investigation. Second, HDR is inherently limited in quiescent tissues such as the adult liver. Addressing the challenges posed by asynchronous cell cycles and the low frequency of S/G2 phases in complex *in vivo* environments is essential for therapeutic efficacy. Notably, our platform's compatibility with AAV-mediated delivery provides a viable path to regulating genome editing outcomes in therapeutic contexts.

MATERIALS AND METHODS

Cell culture

293A (Thermo Fisher Scientific) and HEK293T (ATCC) cells were cultured in Dulbecco's modified Eagle's medium (DMEM) supplemented with 10% fetal bovine serum (FBS) and penicillin/streptomycin at 37°C in an atmosphere of 5% CO_2.

AAV2 production and purification

Confluent HEK293T cells (50%–70%) grown in DMEM supplemented with 5% FBS were transfected with the pHelper vector, pRC2-mi342 vector, and constructed pAAV expression vectors using Polyethyleninmine (PEI) max in T150 flasks. Twenty-four hours after transfection, the medium was changed to DMEM containing 1% FBS; 1% GlutaMAX Supplement (Gibco); 1% 1 M HEPES, pH 7.4 (Thermo Fisher Scientific); and 1%–7.5% sodium bicarbonate (Gibco). After 48 h of culture, the AAV-containing medium was collected, treated with PEG/NaCl solution, and incubated overnight at 4°C. After incubation, the medium was centrifuged (2,500 × *g* for 1 h at 4°C), and the viral pellet was resuspended in Tris-NaCl-EDTA (TNE) buffer. On day 5 post-transfection, the medium and cells were collected and processed separately. Cells were lysed in 0.5 M EDTA (pH 8.0), the homogenates were cleared of debris by centrifugation, and the pH was neutralized using HEPES buffer. AAV2s were precipitated from the lysates and medium using polyethylene glycol (PEG) 8000. The PEG-precipitated AAV2 was collected by centrifugation and then extracted with chloroform. The viral titer of the collected AAV2 was calculated using an AAV Titration Kit (TaKaRa Bio).

AAV transduction

To optimize AAV2 delivery, a functional titration was performed at the *EMX1* locus. Editing efficiency was found to be at a plateau at MOIs above 5.0×10^4 (Figure S2A). Accordingly, an MOI of 1.0×10^4 was used in subsequent experiments to ensure the system remained sensitive to the dynamic range of Acr-mediated inhibition.

For standard experiments, cells seeded in 24-well plates 24 h prior were transduced at an MOI of 1.0×10^4 (1×10^4 cells/well). The AAV2 viral titer was adjusted to 1.0×10^8 genome copies (gc)/μL using TNE buffer, and 1 μL of the viral solution was added to each well. For experiments involving increased vector ratio, the viral titers of AAV2(AcrIIA11+Cdt1/gRNA(EMX1)) or AAV2(AcrIIA11+Cdt1/gRNA(EMX1)/HDR template) were adjusted to 1.0×10^8 (1:1), 2.0×10^8 (1:2), 5.0×10^8 (1:5), and 1.0×10^9 (1:10) gc/μL. These solutions were then co-transduced with 1 μL AAV2(SauCas9) and adjusted to a constant titer of 1.0×10^8 gc/μL.

TIDE analysis

Seventy-two hours after AAV transduction, genomic DNA was extracted using ISOGENOME (Nippon Gene). Genomic DNA (50 ng) was amplified using Tks Gflex DNA polymerase (TaKaRa Bio) with primers (5′-AAACCACCCTTCTCTCTGGC-3′ and 5′-CTCCGAGGAGAAGGCCAAGT-3′) for the EMX1 target site. PCR conditions were 94°C for 1 min for the first denaturation; pre-amplification using 10 cycles of 98°C for 10 s, 70°C–61°C (−1°C per a cycle) for 15 s, and 68°C for 16 s; amplification using 25 cycles of 98°C for 10 s, 61°C for 15 s, and 68°C for 16 s; and a final extension at 68°C for 3 min. PCR amplicons were purified using Ethachinmate (Nippon Gene) and subjected to Sanger sequencing. Sequencing data were analyzed using TIDE.

Restriction enzyme assay and TIDER analysis

293A cells were seeded in 24-well plates. Twenty-four hours after seeding, cells were transduced with AAV vectors. Seventy-two hours later, genomic DNA was extracted using ISOGENOME (Nippon Gene). The target EMX1 site was amplified from 50 ng of genomic DNA using Tks Gflex DNA Polymerase (Takara Bio) with primers (5′-GAGTCTCTAGCAGCGGGTTC-3′ and 5′-CTG TCATTAGCACCGGCAGAA-3′).

The resulting PCR products were purified using Ethachinmate. For restriction enzyme assays, 400 ng of the purified PCR product was digested with EcoRI-HF (New England Biolabs) at 37°C for 1 h and then heat inactivated at 65°C for 30 min. The digested fragments were analyzed using a MultiNA capillary electrophoresis system (Shimadzu). Additionally, indel efficiency and allelic composition were quantified from the Sanger sequencing data using the TIDER program.

Statistical analysis

Statistical significance was analyzed by one-way ANOVA followed by Dunnett's post hoc test using GraphPad Prism 11 (*$p < 0.05$, **$p < 0.005$, ***$p < 0.0005$, ****$p < 0.0001$). Calculated p values are summarized in Table S3.

DATA AND CODE AVAILABILITY

Supplementary information is available for western blotting analysis, MOI determination, and additional genome editing experiments, including off-target evaluation. Furthermore, details regarding plasmid construction, protein sequences, and statistical analysis for all data presented in the graphs are provided in the supplemental information.

ACKNOWLEDGMENTS

This work was supported in part by the Japan Society for the Promotion of Science (JSPS) KAKENHI (JP22H02201, JP20K21253, and 26K01645 to W.N.), JST SPRING (JPMJSP2132 to E.M. and K.K.), JSPS Program for Forming Japan's Peak Research Universities (JSPS J-PEAKS) JPJS00420230011, funding from Takeda Science Foundation (to W.N.), the Naito Foundation (to W.N.), the Uehara Memorial Foundation (to W. N.), the Mochida Memorial Foundation (to W.N.), and the Suzuken Memorial Foundation (to W.N.). We thank Editage for English language editing.

AUTHOR CONTRIBUTIONS

Conceptualization, E.M. and W.N.; data curation, E.M. and K. Nigorikawa; formal analysis, E.M. and K. Nigorikawa; funding acquisition, E.M. and W.N.; investigation, E.M., A.K., K. Nagase and K. Nigorikawa; methodology, E.M. and K. Nigorikawa; resources, E. M. and K. Nigorikawa; supervision, W.N.; validation, E.M. and W.N.; visualization, E.M. and W.N.; writing – original draft, E.M. and W.N.; writing – review and editing, E.M. and W.N.

DECLARATION OF INTERESTS

The authors declare that they have no competing financial interests or personal relationships that could have appeared to influence the work reported in this paper.

SUPPLEMENTAL INFORMATION

Supplemental information can be found online at https://doi.org/10.1016/j.omta.2026.201751.

REFERENCES

1. Matsumoto, D., Tamamura, H., and Nomura, W. (2020). A cell cycle-dependent CRISPR-Cas9 activation system based on an anti-CRISPR protein shows improved genome editing accuracy. Commun. Biol. *3*, 601.

2. Sakaue-Sawano, A., Kurokawa, H., Morimura, T., Hanyu, A., Hama, H., Osawa, H., Kashiwagi, S., Fukami, K., Miyata, T., Miyoshi, H., et al. (2008). Visualizing spatiotemporal dynamics of multicellular cell-cycle progression. Cell *132*, 487–498.

3. Sakaue-Sawano, A., Yo, M., Komatsu, N., Hiratsuka, T., Kogure, T., Hoshida, T., Goshima, N., Matsuda, M., Miyoshi, H., and Miyawaki, A. (2017). Genetically Encoded Tools for Optical Dissection of the Mammalian Cell Cycle. Mol. Cell *68*, 626–640.e5.

4. Matsumoto, D., Kishi, K., Matsugi, E., Inoue, Y., Nigorikawa, K., and Nomura, W. (2023). Cas9-Geminin and Cdt1-fused anti-CRISPR protein synergistically increase editing accuracy. FEBS Lett. *597*, 985–994.

5. Matsumoto, D., Matsugi, E., Kishi, K., Inoue, Y., Nigorikawa, K., and Nomura, W. (2024). SpCas9-HF1 enhances accuracy of cell cycle-dependent genome editing by increasing HDR efficiency, and by reducing off-target effects and indel rates. Mol. Ther. Nucleic Acids *35*, 102124.

6. Matsumoto, D., Kubota, K., Sato, Y., Kato-Inui, T., Nigorikawa, K., Miyaoka, Y., and Nomura, W. (2025). Screening strategy to identify Cas9 variants with higher HDR activity based on diphtheria toxin. J. Biomed. Sci. *32*, 102.

7. Kishi, K., Nigorikawa, K., Hasegawa, Y., Ohta, Y., Matsugi, E., Matsumoto, D., and Nomura, W. (2025). Cell cycle-dependent regulation of CRISPR-Cas9 repetitive activation by anti-CRISPR and Cdt1 fusion in the CRISPRa system. FEBS Lett. *599*, 828–837.

8. Dong, D., Guo, M., Wang, S., Zhu, Y., Wang, S., Xiong, Z., Yang, J., Xu, Z., and Huang, Z. (2017). Structural basis of CRISPR-SpyCas9 inhibition by an anti-CRISPR protein. Nature *546*, 436–439.

9. Harrington, L.B., Doxzen, K.W., Ma, E., Liu, J.J., Knott, G.J., Edraki, A., Garcia, B., Amrani, N., Chen, J.S., Cofsky, J.C., et al. (2017). A Broad-Spectrum Inhibitor of CRISPR-Cas9. Cell *170*, 1224–1233.e15.

10. Meacham, Z., de Tacca, L.A., Bondy-Denomy, J., Rabuka, D., and Schelle, M. (2023). Cas9 degradation in human cells using phage anti-CRISPR proteins. PLoS Biol. *21*, e3002431.

11. Song, G., Zhang, F., Zhang, X., Gao, X., Zhu, X., Fan, D., and Tian, Y. (2019). AcrIIA5 inhibits a broad range of Cas9 orthologs by preventing DNA target cleavage. Cell Rep. *29*, 2579–2589.e4.

12. Yang, H., and Patel, D.J. (2017). Inhibition mechanism of an anti-CRISPR suppressor AcrIIA4 targeting SpyCas9. Mol. Cell *67*, 117–127.e5.

13. Eitzinger, S., Asif, A., Watters, K.E., Iavarone, A.T., Knott, G.J., Doudna, J.A., and Minhas, F.U.A.A. (2020). Machine learning predicts new anti-CRISPR proteins. Nucleic Acids Res. *48*, 4698–4708.

14. Forsberg, K.J., Bhatt, I.V., Schmidtke, D.T., Javanmardi, K., Dillard, K.E., Stoddard, B.L., Finkelstein, I.J., Kaiser, B.K., and Malik, H.S. (2019). Functional metagenomics-guided discovery of potent Cas9 inhibitors in the human microbiome. eLife *8*, e46540.

15. Hynes, A.P., Rousseau, G.M., Agudelo, D., Goulet, A., Amigues, B., Loehr, J., Romero, D.A., Fremaux, C., Horvath, P., Doyon, Y., et al. (2018). Widespread anti-CRISPR proteins in virulent bacteriophages inhibit a range of Cas9 proteins. Nat. Commun. *9*, 2919.

16. Hynes, A.P., Rousseau, G.M., Lemay, M.L., Horvath, P., Romero, D.A., Fremaux, C., and Moineau, S. (2017). An anti-CRISPR from a virulent streptococcal phage inhibits Cas9. Nat. Microbiol. *2*, 1374–1380.

17. Mahendra, C., Christie, K.A., Osuna, B.A., Pinilla-Redondo, R., Kleinstiver, B.P., and Bondy-Denomy, J. (2020). Broad-spectrum anti-CRISPR proteins facilitate horizontal gene transfer. Nat. Microbiol. *5*, 620–629.

18. Pawluk, A., Amrani, N., Zhang, Y., Garcia, B., Hidalgo-Reyes, Y., Lee, J., Edraki, A., Shah, M., Sontheimer, E.J., Maxwell, K.L., and Davidson, A.R. (2016). Naturally occurring off-switches for CRISPR-Cas9. Cell *167*, 1829–1838.e9.

19. Rauch, B.J., Silvis, M.R., Hultquist, J.F., Waters, C.S., McGregor, M.J., Krogan, N.J., and Bondy-Denomy, J. (2017). Inhibition of CRISPR-Cas9 with bacteriophage proteins. Cell *168*, 150–158.e10.

20. Watters, K.E., Shivram, H., Fellmann, C., Lew, R.J., McMahon, B., and Doudna, J.A. (2020). Potent CRISPR-Cas9 inhibitors from genomes. Proc. Natl. Acad. Sci. USA *117*, 6531–6539.

21. Marino, N.D., Pinilla-Redondo, R., Csörgő, B., and Bondy-Denomy, J. (2020). Anti-CRISPR protein applications: natural brakes for CRISPR-Cas technologies. Nat. Methods *17*, 471–479.

22. Pryadkina, M., Lostal, W., Bourg, N., Charton, K., Roudaut, C., Hirsch, M.L., and Richard, I. (2015). A comparison of AAV strategies distinguishes overlapping vectors for efficient systemic delivery of the 6.2 kb Dysferlin coding sequence. Mol. Ther. Methods Clin. Dev. *2*, 15009.

23. Hirsch, M.L., Agbandje-McKenna, M., and Samulski, R.J. (2010). Little vector, big gene transduction: fragmented genome reassembly of adeno-associated virus. Mol. Ther. *18*, 6–8.

24. Wu, Z., Yang, H., and Colosi, P. (2010). Effect of genome size on AAV vector packaging. Mol. Ther. *18*, 80–86.

25. Fuchsbauer, O., Swuec, P., Zimberger, C., Amigues, B., Levesque, S., Agudelo, D., Duringer, A., Chaves-Sanjuan, A., Spinelli, S., Rousseau, G.M., et al. (2019). Cas9 allosteric inhibition by the anti-CRISPR protein AcrIIA6. Mol. Cell *76*, 922–937.e7.

26. Dillard, K.E., Zhang, H., Dubbs, L.Z., Chou, C.W., Terrace, C., Javanmardi, K., Kim, W., Forsberg, K.J., and Finkelstein, I.J. (2025). Mechanism of Cas9 inhibition by AcrIIA11. Nucleic Acids Res. *53*, gkaf318.

27. Liu, H., Zhu, Y., Lu, Z., and Huang, Z. (2021). Structural basis of Staphylococcus aureus Cas9 inhibition by AcrIIA14. Nucleic Acids Res. *49*, 6587–6595.

28. Deng, X., Sun, W., Li, X., Wang, J., Cheng, Z., Sheng, G., and Wang, Y. (2024). An anti-CRISPR that represses its own transcription while blocking Cas9-target DNA binding. Nat. Commun. *15*, 1806.

29. Tan, Y., Chu, A.H.Y., Bao, S., Hoang, D.A., Kebede, F.T., Xiong, W., Ji, M., Shi, J., and Zheng, Z. (2019). Rationally engineered Staphylococcus aureus Cas9 nucleases with high genome-wide specificity. Proc. Natl. Acad. Sci. USA *116*, 20969–20976.

30. Garcia, B., Lee, J., Edraki, A., Hidalgo-Reyes, Y., Erwood, S., Mir, A., Trost, C.N., Seroussi, U., Stanley, S.Y., Cohn, R.D., et al. (2019). Anti-CRISPR AcrIIA5 potently inhibits all Cas9 homologs used for genome editing. Cell Rep. *29*, 1739–1746.e5.

31. Ran, F.A., Cong, L., Yan, W.X., Scott, D.A., Gootenberg, J.S., Kriz, A.J., Zetsche, B., Shalem, O., Wu, X., Makarova, K.S., et al. (2015). In vivo genome editing using Staphylococcus aureus Cas9. Nature *520*, 186–191.

32. Brinkman, E.K., Chen, T., Amendola, M., and van Steensel, B. (2014). Easy quantitative assessment of genome editing by sequence trace decomposition. Nucleic Acids Res. *42*, e168.

33. Ibraheim, R., Tai, P.W.L., Mir, A., Javeed, N., Wang, J., Rodríguez, T.C., Namkung, S., Nelson, S., Khokhar, E.S., Mintzer, E., et al. (2021). Self-inactivating, all-in-one AAV vectors for precision Cas9 genome editing via homology-directed repair in vivo. Nat. Commun. *12*, 6267.

34. Rogers, G.L., Chen, H.Y., Morales, H., and Cannon, P.M. (2019). Homologous recombination-based genome editing by clade F AAVs is inefficient in the absence of a targeted DNA break. Mol. Ther. *27*, 1726–1736.

OMTA, Volume *34*

Supplemental information

SauCas9-based cell cycle-dependent genome editing via AAV delivery

Erina Matsugi, Kanae Kishi, Ayane Kishi, Kohei Nagase, Kiyomi Nigorikawa, and Wataru Nomura

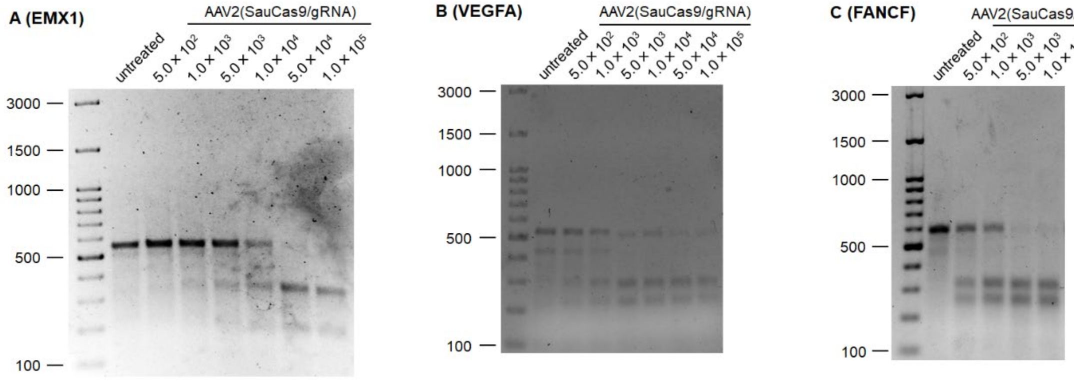

Figure S1. Functional titration of AAV2 for optimization of delivery. Indel efficiency occurred transduction of AAV2(SauCas9/gRNA) with variable viral titers was analyzed by T7E1 analysis on targets EMX1 (A), VEGFA (B), and FANCF (C).

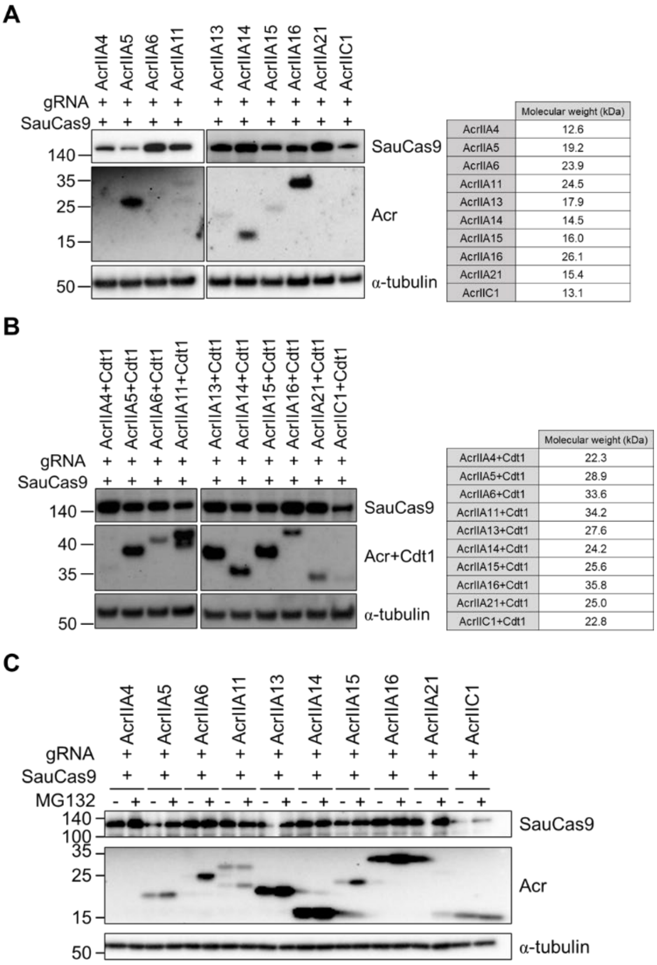

	Molecular weight (kDa)
AcrIIA4	12.6
AcrIIA5	19.2
AcrIIA6	23.9
AcrIIA11	24.5
AcrIIA13	17.9
AcrIIA14	14.5
AcrIIA15	16.0
AcrIIA16	26.1
AcrIIA21	15.4
AcrIIC1	13.1

	Molecular weight (kDa)
AcrIIA4+Cdt1	22.3
AcrIIA5+Cdt1	28.9
AcrIIA6+Cdt1	33.6
AcrIIA11+Cdt1	34.2
AcrIIA13+Cdt1	27.6
AcrIIA14+Cdt1	24.2
AcrIIA15+Cdt1	25.6
AcrIIA16+Cdt1	35.8
AcrIIA21+Cdt1	25.0
AcrIIC1+Cdt1	22.8

Figure S2. Protein expression analysis for Acr candidates (A), and their Cdt1 fusions (B). Cells were infected with the indicated AAVs at an MOI of 1.0×10^4 and collected for analysis at 72 h post-infection. (C) Effect of proteasome inhibition on Acr expression. The effect of the proteasome inhibitor MG132 (3 μM) on the expression of Acr candidates was evaluated.

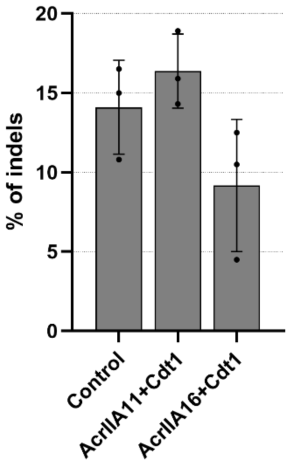

% of indels	Control	AcrIIA11+Cdt1	AcrIIA16+Cdt1
	16.5	15.9	12.5
	15	18.9	10.5
	10.8	14.3	4.5
Average	14.1	16.4	9.2
Standard Deviation	3.0	2.3	4.2
p-value compared with Control		0.6238	0.1861

Figure S3. Quantification of indel efficiency at the EMX1 locus for samples in Figure 4D. The "Control" group represents cells co-transduced with AAV2(SauCas9) and AAV2(gRNA/HDR template). The "AcrIIA11+Cdt1" and "AcrIIA16+Cdt1" groups represent cells co-transduced with AAV2(SauCas9) and either AAV2(AcrIA11+Cdt1/gRNA/HDR template) or AAV2(AcrIIA16+Cdt1/gRNA/HDR template), respectively. Total editing efficiency was quantified by Sanger sequencing followed by TIDER analysis. The indel mutation rate was determined by subtracting the HDR efficiency from the total editing efficiency calculated via the TIDER program. Statistical significance was analyzed by one-way ANOVA followed by Dunnett's post-hoc test.

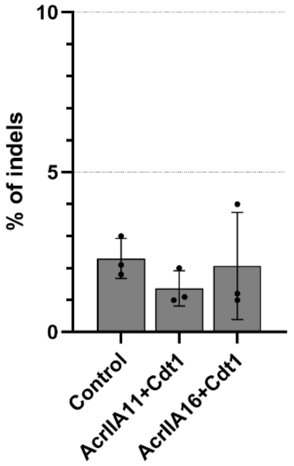

% of indels	Control	AcrIIA11+Cdt1	AcrIIA16+Cdt1
	2.1	2	1.2
	1.8	1	4
	3	1.1	1
Average	2.3	1.4	2.1
Standard Deviation	0.6	0.6	1.7
p-value compared with Control		0.5059	0.9507

Figure S4. Quantification of indel efficiency at the EMX-1 off-target site using the genomic DNA from experiments in Figure 4D. Indel efficiency was quantified by Sanger sequencing followed by TIDE analysis. Off-target locus was amplified by PCR using primers: forward (5'-AACTCCGTCTGTTGATCGGT-3') and reverse (5'-TGCTAGGCTTGAATAACCTGGA-3'). The "Control" group represents cells transduced with AAV2(SauCas9) and AAV2(gRNA/HDR template). The "AcrIIA11+Cdt1" and "AcrIIA16+Cdt1" groups represent cells transduced with AAV2(SauCas9) and either AAV2(AcrIA11+Cdt1/gRNA/HDR template) or AAV2(AcrIIA16+Cdt1/gRNA/HDR template), respectively. Statistical significance was analyzed by one-way ANOVA followed by Dunnett's post-hoc test.

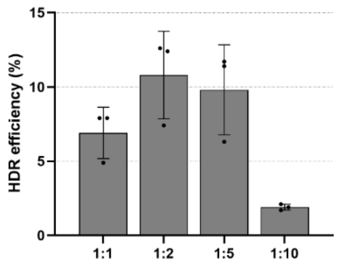

HDR	1:1	1:2	1:5	1:10
	7.9	12.6	11.7	2.1
	7.9	12.4	6.3	1.7
	4.9	7.4	11.4	1.9
Average	6.9	10.8	9.8	1.9
Standard Deviation	1.7	2.9	3.0	0.2
p-value compared with (1:1)		0.16	0.3388	0.0675

Figure S5. Dose-dependent effects of AAV2 carried HDR template on HDR efficiency. The MOI of AAV2(SauCas9) was held constant at $1.0{\times}10^4$, whereas the MOI of AAV2(gRNA/HDR template) was increased as indicated: 1:1 ($1.0{\times}10^4$), 1:2 ($2.0{\times}10^4$), 1:5 ($5.0{\times}10^4$), and 1:10 ($1.0{\times}10^5$). Statistical significance was analyzed by one-way ANOVA followed by Dunnett's post-hoc test.

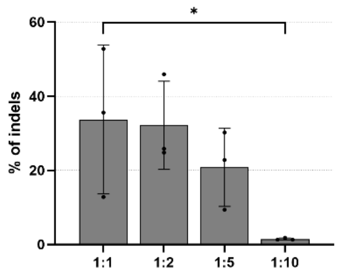

% of indels	1:1	1:2	1:5	1:10
	35.6	25.8	9.4	1.3
	52.8	45.9	22.8	1.8
	12.8	24.8	30.2	1.3
Average	33.7	32.2	20.8	1.5
Standard Deviation	20.1	11.9	10.5	0.3
p-value compared with (1:1)		0.9974	0.5	0.0368

Figure S6. Quantification of indel efficiency at the EMX1 locus for samples in Figure 4E. The MOI of AAV2(SauCas9) was held constant at $1.0{\times}10^4$, whereas the MOI of AAV2(AcrIIA11+Cdt1/gRNA/HDR template) was increased as indicated: 1:1 ($1.0{\times}10^4$), 1:2 ($2.0{\times}10^4$), 1:5 ($5.0{\times}10^4$), and 1:10 ($1.0{\times}10^5$). Total editing efficiency was quantified by Sanger sequencing followed by TIDER analysis. The indel mutation rate was determined by subtracting the HDR efficiency from the total editing efficiency calculated via the TIDER program. Statistical significance was analyzed by one-way ANOVA followed by Dunnett's post-hoc test (*$p < 0.05$).

Table S1 Sequences of synthetic oligonucleotides coding Acr genes.

Anti-CRISPR	Synthetic gene sequence
	SV40NLS, anti-CRISPR, HA
AcrIIA1	CCGAAGAAGAAACGGAAGGTAGGTGGCGGCTTTAATAAGGCTGAAATTATGAAACAAGCTTGGAATTGGTTCAACGATAGCAACATCACGATAAAGCTGTTGGACGAGTTTCTGAAGAAACACGATCTCACACGGTATCAGCTCTCCAAGTTGACAGGGATTAGCCAGAATACTCTCAAAGACCAGAATGAGAAACCTCTCAACAAGTACACCGTCAGTATCCTGAGATCACTGAGTCTGATTAGCGGACTTTCTGTGTCTGATGTGCTGTTCGAACTGGAAGATATCGAGAAGAATTCCGACGATCTTGCTGGCTTCAAACATCTGCTCGACAAATACAAACTGAGCTTTCCAGCACAAGAGTTTGAGCTGTATTGCCTGATAAAGGAGTTCGAATCAGCGAATATCGAGGTACTGCCCTTTACCTTCAACAGGTTCGAGAACGAAGAACACGTTAACATCAAGAAGGACGTCTGTAAAGCCCTGGAAAATGCCATTACTGTGCTGAAAGAGAAGAAGAACGAACTTTTGGGTGGCTCTTACCCCTACGACGTGCCCGACTACGCC
AcrIIA2	CCGAAGAAGAAACGGAAGGTAGGTGGCGGCTTTAATAAGGCTGAAATTATGACGCTTACGCGGGCGCAGAAGAAATACGCGGAGGCAATGCACGAGTTTATCAACATGGTGGATGACTTCGAGGAATCCACACCGGACTTTGCGAAGGAGGTTTTGCACGATAGTGATTACGTAGTCATCACTAAGAACGAAAAGTACGCGGTTGCACTGTGCTCCTTGTCAACAGATGAGTGTGAGTACGATACGAATTTGTATCTTGATGAAAAGTTGGTAGACTATAGCACTGTGGACGTTAACGGAGTCACCTACTACATAAACATCGTTGAGACCAACGATATTGATGATCTTGAGATAGCCACAGATGAAGACGAGATGAAATCAGGAAACCAGGAAATAATCCTTAAGTCTGAACTTGGTGGCTCTTACCCCTACGACGTGCCCGACTACGCC
AcrIIA3	CCGAAGAAGAAACGGAAGGTAGGTGGCGGCTTTAATAAGGCTGAAATTATGTTCAACAAAGCGGAAATAATGAAACAAGCCTGGAATTGGTTTAACGATTCCAATATATGGCTCTCCGATATTGAGTGGGTGTCATACACAGATAAAGAGAAGTCTTTCAGCGTCTGTCTGAAGGCAGCTTGGAGTAAAGCCAAAGAGGAAGTTGAAGAATCTAAAAAGGAGTCTAAACATATTGCAAAATCAGAGGAGCTTAAAGCCTGGAACTGGGCCGAAAGAAAACTTGGCCTCCACTTCAATATTAGCGATGACGAAAAATTTACTTCCGTTAAAGATGAAACGAAGATAAACTTTGGCCTGTCTGTGTGGGCATGCGCCATGAAAGCCGTGAAACTCCATAATGATCTCTTTCCTCAGACCGCCGCAGGTGGCTCTTACCCCTACGACGTGCCCGACTACGCC
AcrIIA4	CCGAAGAAGAAACGGAAGGTAGGTGGCGGCAATATAAATGACCTCATAAGAGAGATAAAGAACAAAGATTACACAGTCAAGCTGAGCGGGACTGACTCCAACAGTATCACTCAACTGATTATCCGCGTCAATAATGATGGGAACGAGTACGTTATTAGTGAAAGCGAAAATGAGTCAATAGTCGAGAAGTTTATCTCCGCTTTTAAGAACGGTTGGAATCAGGAATATGAGGACGAGGAAGAATTTTATAATGACATGCAGACAATAACGCTGAAAAGTGAGCTGAATGGTGGCTCTTACCCCTACGACGTGCCCGACTACGCC
AcrIIA5	CCGAAGAAGAAACGGAAGGTAGGTGGCGGCGCATACGGAAAAAGCAGATACAACTCATACAGGAAACGGAACTTCAGCATTAGCGACAATCAGCGGAGAGAATACGCAAAAAAGATGAAGGAGCTGGAGCAGGCCTTCGAGAACCTGGACGGCTGGTACCTGAGCTCCATGAAGGACTCCGCCTACAAGGATTTTGGCAAGTATGAGATCAGGCTGTCTAACCACAGCGCCGACAATCGGTATCACGATCTGGAGAACGGCAGACTGATCGTGAATGTGAAGGCCTCTAAGCTGAACTTCGTGGACATCATCGAGAATAAGCTGGGCAAGATCATCGAGAAGATCGATACCCTGGACCTGGATAAGTACCGGTTTATCAATGCTACAAAACTGGAGAGAGATATTAAGTGCTATTACAAAGGATACAAAACCAAAAAGGATGTGATTGGTGGCTCTTACCCCTACGACGTGCCCGACTACGCC
AcrIIA6	CCGAAGAAGAAACGGAAGGTAGGTGGCGGCAAGATTAACGATGACATTAAGGAACTGATTCTGGAGTATATGAGCCGCTATTTCAAATTCGAGAACGACTTTTACAAACTGCCTGGCATCAAGTTCACCGACGCCAACTGGCAGAAGTTTAAGAATGGCGGCACAGATATCGAGAAGATGGGAGCAGCAAGGGTGAACGCAATGCTGGACTGCCTGTTCGACGATTTTGAGCTGGCCATGATCGGCAAGGCCCAGACCAACTACTATAACGACAATTCCCTGAAGATGAATATGCCCTTCTACACATACTACGATATGTTCAAGAAGCAGCAGCTGCTGAAGTGGCTGAAGAACAATCGGGACGATGTGATCGGCGGCACCGGCAGAATGTATACAGCCTCTGGCAACTACATCGCCAATGCCTATCTGGAGGTGGCCCTGGAGAGCTCCTCTCTGGGCAGCGGCTCCTACATGCTGCAGATGCGGTTCAAGGATTATAGCAAGGGCCAGGAGCCTATCCCAAGTGGAAGACAGAACAGACTGGAGTGGATTGAAAACAACCTGGAGAACATCAGAGGTGGCTCTTACCCCTACGACGTGCCCGACTACGCC
AcrIIA11	CCGAAGAAGAAACGGAAGGTAGGTGGCGGCGCAGATATGACGCTTCGCCAGTTCTGCGAGCGATATCGCAAGGGTGACTTCCTCGCAAAGGATCGAGAAACTCAAATCGAGGCAGGTTGGTACGATTGGTTTTGTGATGACAAAGCCTTGGCGGGCCGATTGGCAAAAATCTGGGGGATTTTGAAGGGGATAACCTCAGATTATATCTTGGATAACTACCGCGTATGGTTCAAAAACAACTGTCCAATGGTAGGACCACTGTACGACGATGTACGCTTCGAACCGCTTGATGAAGAACAGCGAGATGAGCTCTACTTCGGCGTCGCAATCGACGATAAGAGGAGGGAAAAGAAATACGTCATATTCACTGCTCGAAATGACTATGAAAACGAGTGTGGTTTCAACAACGTGAGAGAAGTACGCCAATTTATAAATGGATGGGAAGACGAATTGAAGAACGAAGAGTTCTATAAGGCTAGGGAGAAAAAACGGCAAGAAATGGAAGAAGCCAATAACAAATTCGCAGAAATAATGCAACGGGCCGATGAGATATTGTGGAACCTGAAAGAGGACGGTGGCTCTTACCCCTACGACGTGCCCGACTACGCC
AcrIIA13	CCGAAGAAGAAACGGAAGGTAGGTGGCGGCATGAAGTCCGTGAAGTACATCAGCAACATGAGCAAGCAAGAGAAAGGCTACCGGGTGTACGTGAACGTGGTCAACGAGGATACCGACAAGGGCTTTCTGTTCCCCAGCGTGCCCAAAGAAGTGATCGAGAACGACAAGATCGACGAGCTGTTCAACTTCGAGCACCACAAGCCTTACGTGCAGAAGGCCAAGAGCAGATACGACAAGAACGGCATCGGCTACAAGATCGTGCAGCTGGACGAGGGCTTCCAGAAGTTCATCGAGCTGAACAAAGAAAAGATGAAGGAAAACCTGGACTACGGTGGCTCTTACCCCTACGACGTGCCCGACTACGCC
AcrIIA14	CCGAAGAAGAAACGGAAGGTAGGTGGCGGCATGAAGTCCGTGAAGTACATCAGCAACATGAGCAAGCAAGAGAAAGGCTACCGGGTGTACGTGAACGTGGTCAACGAGGATACCGACAAGGGCTTTCTGTTCCCCAGCGTGCCCAAAGAAGTGATCGAGAACGACAAGATCGACGAGCTGTTCAACTTCGAGCACCACAAGCCTTACGTGCAGAAGGCCAAGAGCAGATACGACAAGAACGGCATCGGCTACAAGATCGTGCAGCTGGACGAGGGCTTCCAGAAGTTCATCGAGCTGAACAAAGAAAAGATGAAGGAAAACCTGGACTACGGTGGCTCTTACCCCTACGACGTGCCCGACTACGCC
AcrIIA15	CCGAAGAAGAAACGGAAGGTAGGTGGCGGCGTGGATACCTGGATCGTGTACAGAGGCAGAACCGCCGACATGAACAAGAGCTATATCGCCGAGGGCAGCACCTACGAGGAAGTGTACAACAACTTCGTGGATAAGTACGGCTACGACGTGCTGGACGAGGACATCTACGAGATCCAGCTGCTGAAGAAGAACGGCGAGAACCTGGACGACTACGATGTGGACAGCGACGGCATCAACAACTACGACAAGCTGGATGAGTTCCGCGAGAGCGACTACGTGGACCTGGAAGATTACGACTACAGAGAGCTGTTCGAGAACAGCAGCAGCCAGGTGTACTACCACGAGTTCGAGATCACCCACGAGGGTGGCTCTTACCCCTACGACGTGCCCGACTACGCC
AcrIIA16	CCGAAGAAGAAACGGAAGGTAGGTGGCGGCATGGGCTACATCGGCACCAAGAGAAGCGAGAGAAGCCAGGACGCCATCGAGGATTACGAGGTGCCCCTGAACCACTTCAACAAGGACCTGATCCAGGCCTTCATCGACGAGAACGAGGCCTACGACACCCTCAAGACCAAGAAAGTGCGGCTGTGGAAGTTCGTGGCCCCTAGAGCTGGTGCCACAAGCTGGCATCACACCGGCACCTACTACAACAAGACCGACCACTACAGCCTGGAAAAGGTGGCCGATGAGCTGCTGCAGAATGGCGACGAGTGGGAAGAACAGTTCAAGGCCTACGTGAAAGAGGAACAAGAGACAGCTACCAGCGAGCCCGTGTTCCTGAGCGTGATCAAGGTGCAGATCTGGGGCGGCAGCATGAAGAGGCCTAAACTCGTGGGACACGAGGTGGTCATGGGCGTGAAGAAAGAAGGATGGCTGCACGCCGTGTCCAAGGCCACACAGAGCAAGTACAAGCTGAGCGCCAACAAGGTGGAAATGCAGAAGCACTACTCCCTCGAGGACTACAGCGCCCTGACCAAGGACTTCCCCGAGTTCAAAGCCCAGAAGCGGGCCATCAACAAGAAAATGAAGGAAATGTACAACGGTGGCTCTTACCCCTACGACGTGCCCGACTACGCC
AcrIIA21	CCGAAGAAGAAACGGAAGGTAGGTGGCGGCATGGACTACGACAACGAGAACTATCTGATCCCCAAGATCCTGCTGCAGGACGACTTCTACAGCAGCCTGAGCGCCAAGGACATCCTGGTGTATGCCGTGCTGAAGGACAGACAGATCGAGGCCCTGGAAAAAGGCTGGATCGACACCGACGGCAGCATCTACCTGAACTTCAAGCTGATCGAGCTGGCCAAGATGTTCAGCTGCAGCCGGACCACCATGATCGACGTGATGCAGCGGCTGGAAGAAGTGAACCTGATCGAGCGCGAGCGCGTGGACGTGTTCTACGGCTACAGCCTGCCTTACAAGACCTACATCAACGAGGTGGGTGGCTCTTACCCCTACGACGTGCCCGACTACGCC
AcrIIC1	CCGAAGAAGAAACGGAAGGTAGGTGGCGGCATGAAGGAAGTGTTCAAGCTGAAGCCCGAGCTGGTCACCTACAAAGGCTGTGGATGGGCCCTCGCCTGCATCAAGGATGGCGAGATCATCGACCTGACCTACGTCAGAGATCTGGGCATCGAGGAATACGACGAGAACTTCGACGGCCTGGAACCTGAGATCATCTACTACGACGTGGTGGCCAGCCAGGCCTGTAAAGAGGTGGCCTACAGATACGAGGAAATGGGCGAGTTCACCTTCGGCCTGTGCAGCTGCTGGGAGTTCAACGTGATGGGTGGCTCTTACCCCTACGACGTGCCCGACTACGCC

Tables S2 Amino acid sequences of Acr/Acr-Cdt1 constructs, their molecular weight, and accession numbers.

Acr	Molecular weight	Amino acid sequence
AcrIIA4	12609.9	MGGPKKKRKVGGGNINDLIREIKNKDYTVKLSGTDSNSITQLIIRVNNDGNEYVISESENESIVEKFISAFKNGWNQEYEDEEEFYNDMQTITLKSELNGGSYPYDVPDYA
AcrIIA5	19184.8	MGGPKKKRKVGGGAYGKSRYNSYRKRNFSISDNQRREYAKKMKELEQAFENLDGWYLSSMKDSAYKDFGKYEIRLSNHSADNRYHDLENGRLIVNVKASKLNFVDIIENKLGKIIEKIDTLDLDKYRFINATKLERDIKCYYKGYKTKKDVIGGSYPYDVPDYA
AcrIIA6	23876.1	MGGPKKKRKVGGGKINDDIKELILEYMSRYFKFENDFYKLPGIKFTDANWQKFKNGGTDIEKMGAARVNAMLDCLFDDFELAMIGKAQTNYYNDNSLKMNMPFYTYYDMFKKQQLLKWLKNNRDDVIGGTGRMYTASGNYIANAYLEVALESSSLGSGSYMLQMRFKDYSKGQEPIPSGRQNRLEWIENNLENIRGGSYPYDVPDYA
AcrIIA11	24531.5	MGGPKKKRKVGGGADMTLRQFCERYRKGDFLAKDRETQIEAGWYDWFCDDKALAGRLAKIWGILKGITSDYILDNYRVWFKNNCPMVGPLYDDVRFEPLDEEQRDELYFGVAIDDKRREKKYVIFTARNDYENECGFNNVREVRQFINGWEDELKNEEFYKAREKKRQEMEEANNKFAEIMQRADEILWNLKEDGGSYPYDVPDYA
AcrIIA13	17920.8	MGGPKKKRKVGGGMEVMNKSIEIKDQNNIVLIDSLGQFFTDIENDNNGRYNIDYVLLNEVEHDNGNTYYEVGMYRTEEVPFSDKVTQDNVELLEDKWLQIDQQGESYVESIFFENEEDAREYIKLVLKGHETFEETAKAIGVIKGGSYPYDVPDYA
AcrIIA14	14484.5	MGGPKKKRKVGGGMKSVKYISNMSKQEKGYRVYVNVVNEDTDKGFLFPSVPKEVIENDKIDELFNFEHHKPYVQKAKSRYDKNGIGYKIVQLDEGFQKFIELNKEKMKENLDYGGSYPYDVPDYA
AcrIIA15	15954.1	MGGPKKKRKVGGGVDTWIVYRGRTADMNKSYIAEGSTYEEVYNNFVDKYGYDVLDEDIYEIQLLKKNGENLDDYDVDSDGINNYDKLDEFRESDYVDLEDYDYRELFENSSSQVYYHEFEITHEGGSYPYDVPDYA
AcrIIA16	26087.6	MGGPKKKRKVGGGMGYIGTKRSERSQDAIEDYEVPLNHFNKDLIQAFIDENEAYDTLKTKKVRLWKFVAPRAGATSWHHTGTYYNKTDHYSLEKVADELLQNGDEWEEQFKAYVKEEQETATSEPVFLSVIKVQIWGGSMKRPKLVGHEVVMGVKKEGWLHAVSKATQSKYKLSANKVEMQKHYSLEDYSALTKDFPEFKAQKRAINKKMKEMYNGGSYPYDVPDYA
AcrIIA21	15362.6	MGGPKKKRKVGGGMDYDNENYLIPKILLQDDFYSSLSAKDILVYAVLKDRQIEALEKGWIDTDGSIYLNFKLIELAKMFSCSRTTMIDVMQRLEEVNLIERERVDVFYGYSLPYKTYINEVGGSYPYDVPDYA
AcrIIC1	13143.0	MGGPKKKRKVGGGMKEVFKLKPELVTYKGCGWALACIKDGEIIDLTYVRDLGIEEYDENFDGLEPEIIYYDVVASQACKEVAYRYEEMGEFTFGLCSCWEFNVMGGSYPYDVPDYA
AcrIIA4+Cdt1	22293.8	MGGPKKKRKVGGGNINDLIREIKNKDYTVKLSGTDSNSITQLIIRVNNDGNEYVISESENESIVEKFISAFKNGWNQEYEDEEEFYNDMQTITLKSELNGGSYPYDVPDYALEPSPARPALRAPASATSGSRKRARPPAAPGRDQARPPARRRLRLSVDEVSSPSTPEAPDIPACPSPGQKIKKSTPAAGQPPHLTSAQDQDTI
AcrIIA5+Cdt1	28868.7	MGGPKKKRKVGGGAYGKSRYNSYRKRNFSISDNQRREYAKKMKELEQAFENLDGWYLSSMKDSAYKDFGKYEIRLSNHSADNRYHDLENGRLIVNVKASKLNFVDIIENKLGKIIEKIDTLDLDKYRFINATKLERDIKCYYKGYKTKKDVIGGSYPYDVPDYALEPSPARPALRAPASATSGSRKRARPPAAPGRDQARPPARRRLRLSVDEVSSPSTPEAPDIPACPSPGQKIKKSTPAAGQPPHLTSAQDQDTI
AcrIIA6+Cdt1	33560.0	MGGPKKKRKVGGGKINDDIKELILEYMSRYFKFENDFYKLPGIKFTDANWQKFKNGGTDIEKMGAARVNAMLDCLFDDFELAMIGKAQTNYYNDNSLKMNMPFYTYYDMFKKQQLLKWLKNNRDDVIGGTGRMYTASGNYIANAYLEVALESSSLGSGSYMLQMRFKDYSKGQEPIPSGRQNRLEWIENNLENIRGGSYPYDVPDYALEPSPARPALRAPASATSGSRKRARPPAAPGRDQARPPARRRLRLSVDEVSSPSTPEAPDIPACPSPGQKIKKSTPAAGQPPHLTSAQDQDTI
AcrIIA11+Cdt1	34215.4	MGGPKKKRKVGGGADMTLRQFCERYRKGDFLAKDRETQIEAGWYDWFCDDKALAGRLAKIWGILKGITSDYILDNYRVWFKNNCPMVGPLYDDVRFEPLDEEQRDELYFGVAIDDKRREKKYVIFTARNDYENECGFNNVREVRQFINGWEDELKNEEFYKAREKKRQEMEEANNKFAEIMQRADEILWNLKEDGGSYPYDVPDYALEPSPARPALRAPASATSGSRKRARPPAAPGRDQARPPARRRLRLSVDEVSSPSTPEAPDIPACPSPGQKIKKSTPAAGQPPHLTSAQDQDTI
AcrIIA13+Cdt1	27604.7	MGGPKKKRKVGGGMEVMNKSIEIKDQNNIVLIDSLGQFFTDIENDNNGRYNIDYVLLNEVEHDNGNTYYEVGMYRTEEVPFSDKVTQDNVELLEDKWLQIDQQGESYVESIFFENEEDAREYIKLVLKGHETFEETAKAIGVIKGGSYPYDVPDYALEPSPARPALRAPASATSGSRKRARPPAAPGRDQARPPARRRLRLSVDEVSSPSTPEAPDIPACPSPGQKIKKSTPAAGQPPHLTSAQDQDTI
AcrIIA14+Cdt1	24168.4	MGGPKKKRKVGGGMKSVKYISNMSKQEKGYRVYVNVVNEDTDKGFLFPSVPKEVIENDKIDELFNFEHHKPYVQKAKSRYDKNGIGYKIVQLDEGFQKFIELNKEKMKENLDYGGSYPYDVPDYALEPSPARPALRAPASATSGSRKRARPPAAPGRDQARPPARRRLRLSVDEVSSPSTPEAPDIPACPSPGQKIKKSTPAAGQPPHLTSAQDQDTI
AcrIIA15+Cdt1	25638.0	MGGPKKKRKVGGGVDTWIVYRGRTADMNKSYIAEGSTYEEVYNNFVDKYGYDVLDEDIYEIQLLKKNGENLDDYDVDSDGINNYDKLDEFRESDYVDLEDYDYRELFENSSSQVYYHEFEITHEGGSYPYDVPDYALEPSPARPALRAPASATSGSRKRARPPAAPGRDQARPPARRRLRLSVDEVSSPSTPEAPDIPACPSPGQKIKKSTPAAGQPPHLTSAQDQDTI
AcrIIA16+Cdt1	35771.5	MGGPKKKRKVGGGMGYIGTKRSERSQDAIEDYEVPLNHFNKDLIQAFIDENEAYDTLKTKKVRLWKFVAPRAGATSWHHTGTYYNKTDHYSLEKVADELLQNGDEWEEQFKAYVKEEQETATSEPVFLSVIKVQIWGGSMKRPKLVGHEVVMGVKKEGWLHAVSKATQSKYKLSANKVEMQKHYSLEDYSALTKDFPEFKAQKRAINKKMKEMYNGGSYPYDVPDYALEPSPARPALRAPASATSGSRKRARPPAAPGRDQARPPARRRLRLSVDEVSSPSTPEAPDIPACPSPGQKIKKSTPAAGQPPHLTSAQDQDTI
AcrIIA21+Cdt1	25046.5	MGGPKKKRKVGGGMDYDNENYLIPKILLQDDFYSSLSAKDILVYAVLKDRQIEALEKGWIDTDGSIYLNFKLIELAKMFSCSRTTMIDVMQRLEEVNLIERERVDVFYGYSLPYKTYINEVGGSYPYDVPDYALEPSPARPALRAPASATSGSRKRARPPAAPGRDQARPPARRRLRLSVDEVSSPSTPEAPDIPACPSPGQKIKKSTPAAGQPPHLTSAQDQDTI
AcrIIC1+Cdt1	22826.9	MGGPKKKRKVGGGMKEVFKLKPELVTYKGCGWALACIKDGEIIDLTYVRDLGIEEYDENFDGLEPEIIYYDVVASQACKEVAYRYEEMGEFTFGLCSCWEFNVMGGSYPYDVPDYALEPSPARPALRAPASATSGSRKRARPPAAPGRDQARPPARRRLRLSVDEVSSPSTPEAPDIPACPSPGQKIKKSTPAAGQPPHLTSAQDQDTI

Tables S3 The data used to generate the graphs in the corresponding figures.

**Figure 2B**												
% of indels	SauCas9	SauCas9/gRNA	AcrIIA4	AcrIIA5	AcrIIA6	AcrIIA11	AcrIIA13	AcrIIA14	AcrIIA15	AcrIIA16	AcrIIA21	AcrIIC1
	3.7	61.5	71.1	2.4	64.2	62.3	1.5	2.2	7.5	42.6	81.1	14.9
	1.5	73.4	77.3	2.1	73.1	70.7	0	1.4	5.8	69.3	86.9	15.1
	7.5	77.6	76	4.3	46.6	67.9	1.5	5.1	8.3	84.3	71.9	35.3
	0.3	57.2	20	1.5	51.3	85.2	3.2	3.8	8.8	59	75	28.9
	6.1	57.2	35.2	0	11.7	65.4	2.3	4	8.1	70.8		25.5
	2	66.7	80.9	1.6	79	32						
	1.5	44.8	67.7	2.5	65.5	68						
	1.2			1.7		51.1						
	0.8			1.5		44.2						
	1.7			3.4		87.1						
	0.6			2.8		77.1						
	1.9			1.8		59.2						
Average	2.4	62.6	61.2	2.1	55.9	64.2	1.7	3.3	7.7	65.2	78.7	23.9
Standard Deviation	2.2	11.1	23.7	1.1	22.6	16.1	1.2	1.5	1.2	15.5	6.7	8.9
p-value compared with SauCas9/gRNA	N/A		0.943	<0.0001	0.363	0.242	<0.0001	<0.0001	<0.0001	0.0317	0.996	0.0426

**Figure 2C**												
% of indels	SauCas9	SauCas9/gRNA	AcrIIA4+Cdt1	AcrIIA5+Cdt1	AcrIIA6+Cdt1	AcrIIA11+Cdt1	AcrIIA13+Cdt1	AcrIIA14+Cdt1	AcrIIA15+Cdt1	AcrIIA16+Cdt1	AcrIIA21+Cdt1	AcrIIC1+Cdt1
	3	84.3	83.5	3.2	60.9	27.3	40.1	23.2	32.3	65	73.4	57
	1.2	77.3	80.9	11	67.5	42.8	10.6	5.4	10.2	46.6	70.5	45.5
	1.8	36.6	77.8	0.6	41.9	53.7	0.2	0	0	35.8	61.7	44.7
	3.7	72	49.4	34.9	39	69.5	7.7	3.7	4.3	42.8	76.1	46.8
	1.6	60.1	76.1	3.2	50.5	70.1	12.4	8.5	17.2	49.4	85.2	53
	9.8	83.4	85.2	3.9	80.6	60.7	12.5	5	6.4	58	74.8	54.9
	11.2			12		19						
	3.6			3.9		73.1						
	4.4			0.2		19.3						
	0.8			4.5		84						
	5			7.9		83.7						
	1.7			2.7		80.1						
	2.7			4.5		61						
Average	3.9	69.0	75.5	7.1	56.7	57.3	13.9	7.6	11.7	49.6	73.6	50.3
Standard Deviation	3.2	18.2	13.2	9.1	16.0	23.4	13.6	8.1	11.6	10.5	7.7	5.3
p-value compared with SauCas9/gRNA	N/A		>0.999	<0.0001	0.879	>0.999	<0.0001	<0.0001	<0.0001	>0.999	0.13	<0.0001

**Figure 3A**						
% of indels	SauCas9	SauCas9/gRNA	1:1	1:2	1:5	1:10
	1.5	61.5	70.7	67.1	48.2	27.8
	7.5	73.4	67.9	78.8	65.5	57.3
	0.3	77.6	77.6	77.2	68.9	37.9
	6.1	57.2	65.4	54.7	57.1	30.8
Average	3.9	67.4	70.4	69.5	59.9	38.5
Standard Deviation	3.5	9.6	5.3	11.1	9.3	13.3
p-value compared with SauCas9/gRNA	NA		0.988	0.998	0.682	0.0015

**Figure 3B**						
% of indels	SauCas9	SauCas9/gRNA	1:1	1:2	1:5	1:10
	1.5	61.5	77.8	30.2	21.2	9.3
	1.6	77.3	70.1	45.1	5.1	11.3
	9.8	57.2	71.9	9.5	11.2	19.3
Average	4.3	65.3	73.3	28.3	12.5	13.3
Standard Deviation	4.8	10.6	4.0	17.9	8.1	5.3
p-value compared with SauCas9/gRNA	NA		0.774	0.0022	<0.0001	0.0001

**Figure 4D**			
HDR	Control	AcrIIA11+Cdt1	AcrIIA16+Cdt1
	8	12.1	13.8
	5.2	12.5	10.2
	4	13.4	12.3
Average	5.7	12.7	12.1
Standard Deviation	2.1	0.7	1.8
p-value compared with Control		0.0035	0.0054

**Figure 4E**				
HDR	1:1	1:2	1:5	1:10
	10	13.6	15.8	3.8
	11.2	12.8	13.8	2.5
	6.9	16.4	17.5	2.4
Average	9.4	14.3	15.7	2.9
Standard Deviation	2.2	1.9	1.9	0.8
p-value compared with (1:1)		0.0237	0.006	0.0053

**Figure 4F**			
HDR	Control	AcrIIA11+Cdt1	AcrIIA16+Cdt1
	0.3	3.9	1.6
	0.4	3	1.9
	0	2.9	1.2
	0.4	2.2	1.9
Average	0.28	3.00	1.65
Standard Deviation	0.19	0.70	0.33
p-value compared with Control		<0.0001	0.004

Supplemental Methods

Plasmid construction

pAAV_SauCas9_gRNA(EMX1): The experimental procedure was followed for the instruction manual of AAVpro® CRISPR/SaCas9 Helper Free System (AAV2) (Takara Bio). Oligo-1 (EMX1) and Oligo-2 (EMX1) were synthesized by Eurofins Genomics. The fragments were annealed and ligated to pAAV-Guide-it-1 Vector using DNA Ligation Kit＜Mighty Mix＞ (Takara Bio), resulting pAAV_SauCas9_gRNA(EMX1).

Oligo-1(EMX1); 5'-ACCGGCAACCACAAACCCACGAGGG-3'

Oligo-2(EMX1); 5'-AAACCCCTCGTGGGTTTGTGGTTGC-3'

pAAV_SauCas9_gRNA(VEGFA): The plasmid was constructed in the same procedure as pAAV_SauCas9_gRNA (EMX1) using the following Oligo-1 and Oligo-2.

Oligo-1(VEGFA); 5'-ACCGGGGTGAGTGAGTGTGTGCGTG-3'

Oligo-2(VEGFA); 5'-AAACCACGCACACACTCACTCACCC-3'

pAAV_SauCas9_gRNA(FANCF): The plasmid was constructed in the same procedure as pAAV_SauCas9_gRNA (EMX1) using the following Oligo-1 and Oligo-2.

Oligo-1(FANCF); 5'-ACCGGCAAGGCCCGGCGCACGGTGG-3'

Oligo-2(FANCF); 5'-AAACCCACCGTGCGCCGGGCCTTGC-3'

pAAV_SauCas9: pAAV_SauCas9_gRNA(EMX1) was digested with EcoRI/NotI, then blunt end treatment was performed by using Klenow Fragment (Takara Bio). The plasmid was ligated using DNA Ligation Kit<Mighty Mix> (Takara Bio), resulting pAAV_SauCas9.

pAAV_Acrs-Cdt1_gRNA (Acrs, AcrIIA4/A5/A6/A11): DNA fragments for NLS_Acrs_HA (Acrs; AcrIIA4/A5/A6/A11) were synthesized by Eurofins Genomics. Each fragment was PCR amplified with pFucci primers and digested with BamHI/XhoI. The same treatment was done for pFucci-G1 Orange Expression vector (MBL) and the fragments were ligated to the N-terminus of hCdt1(30-120) sequence by using DNA Ligation Kit<Mighty Mix> (Cat# 6023, Takara Bio) resulting pFucci_Acrs-Cdt1. Acrs-Cdt1 sequences were PCR amplified with pAAV Acr primers, and digested with AflII/BamHI. The same treatment was done for pAAV_SauCas9_gRNA, and the fragments were ligated with DNA Ligation Kit<Mighty Mix> (Takara Bio), resulting pAAV_AcrIIA4/A5/A6/A11+Cdt1_gRNA.

pFucci Fw primer; 5'-GAGCTCGGATCCGCCACCATGGGTGGCCCGA-3'

pFucci Rv primer; 5'-ATTATTCTCGAGGGCGTAGTCGGGCACGTC-3'

pAAV Acr Fw primer; 5'-GGAGAAGACTTAAGGCCGCCACCATGGGTGGCCCGAAGAAG-3'

pAAV Acr Rv primer; 5'-CCTCTTCTGGATCCTTAGATGGTGTCCTGGTCCTGCGC-3'

pAAV_Acrs-Cdt1_gRNA (Acrs; AcrIIA13/A14/A15/A16/A21/C1): DNA fragments for NLS_Acrs_HA (Acrs; AcrIIA13/A14/A15/A16/A21/C1) were synthesized by Eurofins Genomics.

Each fragments were PCR amplified by pAAV_AcrIIA13-A16/A21/C1+Cdt1_gRNA primers and the fragments were assembled with AflII/XhoI digested pAAV_AcrIIA5+Cdt1_gRNA(EMX1) using NEBuilder® HiFi DNA Assembly Master Mix (New England Biolabs), resulting pAAV_AcrIIA13/A14/A15/A16/A21/C1+Cdt1_gRNA.
pAAV_AcrIIA13-A16/A21/C1+Cdt1_gRNA Fw;
5'-AGCTGGTTTAGTGGATATCCTTAAGGC-3'
pAAV_AcrIIA13-A16/A21/C1+Cdt1_gRNA Rv;
5'-CGGGCCTGGCGGGGCTGGGCTCGAGGGCGTAGTCGGGCACGTCGT-3'

pAAV_Acrs_gRNA:

Acr genes were amplified by PCR using pAAV_Acrs-Cdt1_gRNA plasmids as templates and pAAV_Acr_gRNA primers. The fragments were assembled with AflII/BamHI digested pAAV_AcrIIA11+Cdt1_gRNA(EMX1) using NEBuilder® HiFi DNA Assembly Master Mix (New England Biolabs), resulting pAAV_Acrs_gRNA (Acrs; AcrIIA4/A5/A6/A11/ A13/A14/A15/A16/A21/C1).
pAAV_Acr_gRNA Fw;
5'-AGCTGGTTTAGTGGATATCCTTAAGGC-3'
pAAV_Acr_gRNA Rv;
5'-TGGCAACTAGAAGGCACAGGGATCCTTAGGCGTAGTCGGGCACGTCGT-3'

pAAV_Acrs-Cdt1_gRNA_HDR template (Acrs; AcrIIA5, A11, A13, A14, A15, A16, C1):

5' and 3' homology arm (HA) fragments were amplified by PCR with EMX1 5' HA primers and EMX1 3' HA primers, respectively, using genomic DNA extracted from 293A cells. The amplified HA fragments were assembled using NEBuilder® HiFi DNA Assembly Master Mix (New England Biolabs), resulting the HDR template fragment. The fragment was inserted to NotI digested pAAV_Acrs-Cdt1_gRNA using genomic DNA extracted from 293A cells. The amplified HA fragments were assembled using NEBuilder® HiFi DNA Assembly Master Mix (New England Biolabs), resulting pAAV_Acrs-Cdt1_gRNA(EMX1)_HDR template.
EMX1 5'-HA Fw; 5'-ACTTGTTGGCGAGATTTTTGCCTCTTGATATAAACACCACCTC-3'
EMX1 5'-HA Rv; 5'-CACCCTAGAATTCGTCATTGGAGGTGACATCGA-3'
EMX1 3'-HA Fw; 5'-CAATGACGAATTCTAGGGTGGGCAACCACAAAC-3'
EMX1 3'-HA Rv; 5'-CTAGGGGTTCCTAGATCTGCTCTAGGCAGAGGGAAGCACT-3'

pAAV_gRNA_HDR template:

pAAV_AcrIIA5+Cdt1_gRNA_HDR template was digested with AflII/EcoRI to remove AcrIIA5+Cdt1 gene and the polyA sequence. Oligonucleotides including T7 &T3 promoter sequences were synthesized by Eurofins Genomics. The oligonucleotides were annealed and resulting double-stranded fragment was ligated to digested pAAV_AcrIIA5+Cdt1_gRNA_HDR template using DNA Ligation mix<Mighty Mix> (Takara Bio), resulting pAAV_gRNA_HDR template.

pAAV_T7&T3_1;
5'-TTAAGTAATACGACTCACTATAGGGGCAATTAACCCTCACTAAAGGGGTACCG-3'
pAAV_T7&T3_2;
5'-AATTCGGTACCCCTTTAGTGAGGGTTAATTGCCCCTATAGTGAGTCGTATTAC-3'

Western blotting

Proteins within each sample were separated by SDS-PAGE using either the NuPAGE electrophoresis system (Thermo Fisher) (Figure S1A and S1B) or the standard Laemmli's method (Figure S1C), then transferred to nitrocellulose membranes. The membranes were blocked with 4% skim milk in TBS-T (0.1% Tween 20 in Tris-buffered saline) for 30 min, followed by incubation with the primary antibody. After washing with TBS-T, the membranes were incubated with the appropriate horseradish peroxidase-conjugated secondary antibody, and immunoreactivity was detected using ECL detection reagents. For the detection of SauCas9 and Acrs, mouse monoclonal anti-FLAG (A8592, Sigma-Aldrich) and rat monoclonal anti-HA (12158167001, Sigma-Aldrich) were used as primary antibodies, respectively. Anti-rat IgG HRP-linked antibody (ab97057, Abcam) and anti-rabbit IgG HRP-linked antibody (#7074, Cell Signaling Technology) were used as secondary antibodies. Polyclonal anti-α-tubulin (PM054, MBL Life Science) was used as a loading control.

T7E1 assay

293A cells were seeded in 12-well plates at a density of 1.0×10^5 cells/well. Twenty-four hours post-seeding, the cells were infected with AAV2(SauCas9/gRNA) at MOIs of 5.0×10^2, 1.0×10^3, 5.0×10^3, 1.0×10^4, 5.0×10^4, and 1.0×10^5, except for the FANCF site (5.0×10^2, 1.0×10^3, 5.0×10^3, 1.0×10^4). The titers of AAV2 were 5.4×10^8 gc/μL for AAV2(SauCas9/gRNA(EMX1)), 5.3×10^8 gc/μL for AAV2(SauCas9/gRNA(VEGFA)), and 1.0×10^8 gc/μL for AAV2(SauCas9/gRNA(FANCF)). The viral titer was adjusted to 1.0×10^8 gc/μL with TNE buffer and the calculated specific viral load was applied directly. Forty-two hours post-infection, half of the proliferated cells were passaged into a new 12-well plate. Genomic DNA was extracted 24 hours later using the QIAamp DNA Mini Kit (QIAGEN). The target regions were PCR-amplified using Tks Gflex™ DNA Polymerase (Takara Bio), followed by purification using the MinElute PCR Purification Kit (Qiagen).
The following primer pairs were used for PCR;
EMX1;
Fw (5'-GGAGCAGCTGGTCAGAGGGG-3') and Rv (5'-GGGAAGGGGGACACTGGGGA-3')
VEGFA;
Fw (5'-AGATGGCACATTGTCAGAGG-3') and Rv (5'-GAAACTCTGTCCAGAGACACG-3')
FANCF;
Fw (5'-GGGCCGGGAAAGAGTTGCTG-3') and Rv (5'-GCCCTACATCTGCTCTCCCTCC-3')

For the T7E1 assay, 200 ng of the amplified DNA fragment and 1 μL of 10× NEBuffer 2 (New England Biolabs) were mixed with H_2O to a total volume of 9 μL. Re-annealing was performed by heating at 95°C for 5 min, cooling to 85°C at -2°C/s, and further cooling to 25°C at -0.1°C/s, followed by holding at 4°C. After annealing, 1 μL of T7 Endonuclease I (New England Biolabs) was added, and the mixture was incubated at 37°C for 1 hour. The resulting DNA fragments were analyzed via electrophoresis.
